# Measurements of the $\mathrm{t}\overline{\mathrm{t}}$ production cross section in lepton+jets final states in pp collisions at 8 $\,\text {TeV}$ and ratio of 8 to 7 $\,\text {TeV}$ cross sections

**DOI:** 10.1140/epjc/s10052-016-4504-z

**Published:** 2017-01-07

**Authors:** V. Khachatryan, A. M. Sirunyan, A. Tumasyan, W. Adam, E. Asilar, T. Bergauer, J. Brandstetter, E. Brondolin, M. Dragicevic, J. Erö, M. Flechl, M. Friedl, R. Frühwirth, V. M. Ghete, C. Hartl, N. Hörmann, J. Hrubec, M. Jeitler, V. Knünz, A. König, M. Krammer, I. Krätschmer, D. Liko, T. Matsushita, I. Mikulec, D. Rabady, B. Rahbaran, H. Rohringer, J. Schieck, R. Schöfbeck, J. Strauss, W. Treberer-Treberspurg, W. Waltenberger, C.-E. Wulz, V. Mossolov, N. Shumeiko, J. Suarez Gonzalez, S. Alderweireldt, T. Cornelis, E. A. De Wolf, X. Janssen, A. Knutsson, J. Lauwers, S. Luyckx, M. Van De Klundert, H. Van Haevermaet, P. Van Mechelen, N. Van Remortel, A. Van Spilbeeck, S. Abu Zeid, F. Blekman, J. D’Hondt, N. Daci, I. De Bruyn, K. Deroover, N. Heracleous, J. Keaveney, S. Lowette, M. Maes, L. Moreels, A. Olbrechts, Q. Python, D. Strom, S. Tavernier, W. Van Doninck, P. Van Mulders, G. P. Van Onsem, I. Van Parijs, P. Barria, H. Brun, C. Caillol, B. Clerbaux, G. De Lentdecker, G. Fasanella, L. Favart, A. Grebenyuk, G. Karapostoli, T. Lenzi, A. Léonard, T. Maerschalk, A. Marinov, L. Perniè, A. Randle-conde, T. Reis, T. Seva, C. Vander Velde, P. Vanlaer, R. Yonamine, F. Zenoni, F. Zhang, K. Beernaert, L. Benucci, A. Cimmino, S. Costantini, S. Crucy, D. Dobur, A. Fagot, G. Garcia, M. Gul, J. Mccartin, A. A. Ocampo Rios, D. Poyraz, D. Ryckbosch, S. Salva, M. Sigamani, N. Strobbe, M. Tytgat, W. Van Driessche, E. Yazgan, N. Zaganidis, S. Basegmez, C. Beluffi, O. Bondu, S. Brochet, G. Bruno, A. Caudron, L. Ceard, G. G. Da Silveira, C. Delaere, D. Favart, L. Forthomme, A. Giammanco, J. Hollar, A. Jafari, P. Jez, M. Komm, V. Lemaitre, A. Mertens, M. Musich, C. Nuttens, L. Perrini, A. Pin, K. Piotrzkowski, A. Popov, L. Quertenmont, M. Selvaggi, M. Vidal Marono, N. Beliy, G. H. Hammad, W. L. Aldá Júnior, F. L. Alves, G. A. Alves, L. Brito, M. Correa Martins Junior, M. Hamer, C. Hensel, C. Mora Herrera, A. Moraes, M. E. Pol, P. Rebello Teles, E. Belchior Batista Das Chagas, W. Carvalho, J. Chinellato, A. Custódio, E. M. Da Costa, D. De Jesus Damiao, C. De Oliveira Martins, S. Fonseca De Souza, L. M. Huertas Guativa, H. Malbouisson, D. Matos Figueiredo, L. Mundim, H. Nogima, W. L. Prado Da Silva, A. Santoro, A. Sznajder, E. J. Tonelli Manganote, A. Vilela Pereira, S. Ahuja, C. A. Bernardes, A. De Souza Santos, S. Dogra, T. R. Fernandez Perez Tomei, E. M. Gregores, P. G. Mercadante, C. S. Moon, S. F. Novaes, Sandra S. Padula, D. Romero Abad, J. C. Ruiz Vargas, A. Aleksandrov, R. Hadjiiska, P. Iaydjiev, M. Rodozov, S. Stoykova, G. Sultanov, M. Vutova, A. Dimitrov, I. Glushkov, L. Litov, B. Pavlov, P. Petkov, M. Ahmad, J. G. Bian, G. M. Chen, H. S. Chen, M. Chen, T. Cheng, R. Du, C. H. Jiang, R. Plestina, F. Romeo, S. M. Shaheen, A. Spiezia, J. Tao, C. Wang, Z. Wang, H. Zhang, C. Asawatangtrakuldee, Y. Ban, Q. Li, S. Liu, Y. Mao, S. J. Qian, D. Wang, Z. Xu, C. Avila, A. Cabrera, L. F. Chaparro Sierra, C. Florez, J. P. Gomez, B. Gomez Moreno, J. C. Sanabria, N. Godinovic, D. Lelas, I. Puljak, P. M. Ribeiro Cipriano, Z. Antunovic, M. Kovac, V. Brigljevic, K. Kadija, J. Luetic, S. Micanovic, L. Sudic, A. Attikis, G. Mavromanolakis, J. Mousa, C. Nicolaou, F. Ptochos, P. A. Razis, H. Rykaczewski, M. Bodlak, M. Finger, M. Finger, A. A. Abdelalim, A. Awad, M. El Sawy, A. Mahrous, A. Radi, B. Calpas, M. Kadastik, M. Murumaa, M. Raidal, A. Tiko, C. Veelken, P. Eerola, J. Pekkanen, M. Voutilainen, J. Härkönen, V. Karimäki, R. Kinnunen, T. Lampén, K. Lassila-Perini, S. Lehti, T. Lindén, P. Luukka, T. Mäenpää, T. Peltola, E. Tuominen, J. Tuominiemi, E. Tuovinen, L. Wendland, J. Talvitie, T. Tuuva, M. Besancon, F. Couderc, M. Dejardin, D. Denegri, B. Fabbro, J. L. Faure, C. Favaro, F. Ferri, S. Ganjour, A. Givernaud, P. Gras, G. Hamel de Monchenault, P. Jarry, E. Locci, M. Machet, J. Malcles, J. Rander, A. Rosowsky, M. Titov, A. Zghiche, I. Antropov, S. Baffioni, F. Beaudette, P. Busson, L. Cadamuro, E. Chapon, C. Charlot, T. Dahms, O. Davignon, N. Filipovic, A. Florent, R. Granier de Cassagnac, S. Lisniak, L. Mastrolorenzo, P. Miné, I. N. Naranjo, M. Nguyen, C. Ochando, G. Ortona, P. Paganini, P. Pigard, S. Regnard, R. Salerno, J. B. Sauvan, Y. Sirois, T. Strebler, Y. Yilmaz, A. Zabi, J.-L. Agram, J. Andrea, A. Aubin, D. Bloch, J.-M. Brom, M. Buttignol, E. C. Chabert, N. Chanon, C. Collard, E. Conte, X. Coubez, J.-C. Fontaine, D. Gelé, U. Goerlach, C. Goetzmann, A.-C. Le Bihan, J. A. Merlin, K. Skovpen, P. Van Hove, S. Gadrat, S. Beauceron, C. Bernet, G. Boudoul, E. Bouvier, C. A. Carrillo Montoya, R. Chierici, D. Contardo, B. Courbon, P. Depasse, H. El Mamouni, J. Fan, J. Fay, S. Gascon, M. Gouzevitch, B. Ille, F. Lagarde, I. B. Laktineh, M. Lethuillier, L. Mirabito, A. L. Pequegnot, S. Perries, J. D. Ruiz Alvarez, D. Sabes, L. Sgandurra, V. Sordini, M. Vander Donckt, P. Verdier, S. Viret, T. Toriashvili, Z. Tsamalaidze, C. Autermann, S. Beranek, M. Edelhoff, L. Feld, A. Heister, M. K. Kiesel, K. Klein, M. Lipinski, A. Ostapchuk, M. Preuten, F. Raupach, S. Schael, J. F. Schulte, T. Verlage, H. Weber, B. Wittmer, V. Zhukov, M. Ata, M. Brodski, E. Dietz-Laursonn, D. Duchardt, M. Endres, M. Erdmann, S. Erdweg, T. Esch, R. Fischer, A. Güth, T. Hebbeker, C. Heidemann, K. Hoepfner, D. Klingebiel, S. Knutzen, P. Kreuzer, M. Merschmeyer, A. Meyer, P. Millet, M. Olschewski, K. Padeken, P. Papacz, T. Pook, M. Radziej, H. Reithler, M. Rieger, F. Scheuch, L. Sonnenschein, D. Teyssier, S. Thüer, V. Cherepanov, Y. Erdogan, G. Flügge, H. Geenen, M. Geisler, F. Hoehle, B. Kargoll, T. Kress, Y. Kuessel, A. Künsken, J. Lingemann, A. Nehrkorn, A. Nowack, I. M. Nugent, C. Pistone, O. Pooth, A. Stahl, M. Aldaya Martin, I. Asin, N. Bartosik, O. Behnke, U. Behrens, A. J. Bell, K. Borras, A. Burgmeier, A. Campbell, S. Choudhury, F. Costanza, C. Diez Pardos, G. Dolinska, S. Dooling, T. Dorland, G. Eckerlin, D. Eckstein, T. Eichhorn, G. Flucke, E. Gallo, J. Garay Garcia, A. Geiser, A. Gizhko, P. Gunnellini, J. Hauk, M. Hempel, H. Jung, A. Kalogeropoulos, O. Karacheban, M. Kasemann, P. Katsas, J. Kieseler, C. Kleinwort, I. Korol, W. Lange, J. Leonard, K. Lipka, A. Lobanov, W. Lohmann, R. Mankel, I. Marfin, I.-A. Melzer-Pellmann, A. B. Meyer, G. Mittag, J. Mnich, A. Mussgiller, S. Naumann-Emme, A. Nayak, E. Ntomari, H. Perrey, D. Pitzl, R. Placakyte, A. Raspereza, B. Roland, M. Ö. Sahin, P. Saxena, T. Schoerner-Sadenius, M. Schröder, C. Seitz, S. Spannagel, K. D. Trippkewitz, R. Walsh, C. Wissing, V. Blobel, M. Centis Vignali, A. R. Draeger, J. Erfle, E. Garutti, K. Goebel, D. Gonzalez, M. Görner, J. Haller, M. Hoffmann, R. S. Höing, A. Junkes, R. Klanner, R. Kogler, N. Kovalchuk, T. Lapsien, T. Lenz, I. Marchesini, D. Marconi, M. Meyer, D. Nowatschin, J. Ott, F. Pantaleo, T. Peiffer, A. Perieanu, N. Pietsch, J. Poehlsen, D. Rathjens, C. Sander, C. Scharf, H. Schettler, P. Schleper, E. Schlieckau, A. Schmidt, J. Schwandt, V. Sola, H. Stadie, G. Steinbrück, H. Tholen, D. Troendle, E. Usai, L. Vanelderen, A. Vanhoefer, B. Vormwald, M. Akbiyik, C. Barth, C. Baus, J. Berger, C. Böser, E. Butz, T. Chwalek, F. Colombo, W. De Boer, A. Descroix, A. Dierlamm, S. Fink, F. Frensch, R. Friese, M. Giffels, A. Gilbert, D. Haitz, F. Hartmann, S. M. Heindl, U. Husemann, I. Katkov, A. Kornmayer, P. Lobelle Pardo, B. Maier, H. Mildner, M. U. Mozer, T. Müller, Th. Müller, M. Plagge, G. Quast, K. Rabbertz, S. Röcker, F. Roscher, G. Sieber, H. J. Simonis, F. M. Stober, R. Ulrich, J. Wagner-Kuhr, S. Wayand, M. Weber, T. Weiler, C. Wöhrmann, R. Wolf, G. Anagnostou, G. Daskalakis, T. Geralis, V. A. Giakoumopoulou, A. Kyriakis, D. Loukas, A. Psallidas, I. Topsis-Giotis, A. Agapitos, S. Kesisoglou, A. Panagiotou, N. Saoulidou, E. Tziaferi, I. Evangelou, G. Flouris, C. Foudas, P. Kokkas, N. Loukas, N. Manthos, I. Papadopoulos, E. Paradas, J. Strologas, G. Bencze, C. Hajdu, A. Hazi, P. Hidas, D. Horvath, F. Sikler, V. Veszpremi, G. Vesztergombi, A. J. Zsigmond, N. Beni, S. Czellar, J. Karancsi, J. Molnar, Z. Szillasi, M. Bartók, A. Makovec, P. Raics, Z. L. Trocsanyi, B. Ujvari, P. Mal, K. Mandal, D. K. Sahoo, N. Sahoo, S. K. Swain, S. Bansal, S. B. Beri, V. Bhatnagar, R. Chawla, R. Gupta, U. Bhawandeep, A. K. Kalsi, A. Kaur, M. Kaur, R. Kumar, A. Mehta, M. Mittal, J. B. Singh, G. Walia, Ashok Kumar, A. Bhardwaj, B. C. Choudhary, R. B. Garg, A. Kumar, S. Malhotra, M. Naimuddin, N. Nishu, K. Ranjan, R. Sharma, V. Sharma, S. Bhattacharya, K. Chatterjee, S. Dey, S. Dutta, Sa. Jain, N. Majumdar, A. Modak, K. Mondal, S. Mukherjee, S. Mukhopadhyay, A. Roy, D. Roy, S. Roy Chowdhury, S. Sarkar, M. Sharan, A. Abdulsalam, R. Chudasama, D. Dutta, V. Jha, V. Kumar, A. K. Mohanty, L. M. Pant, P. Shukla, A. Topkar, T. Aziz, S. Banerjee, S. Bhowmik, R. M. Chatterjee, R. K. Dewanjee, S. Dugad, S. Ganguly, S. Ghosh, M. Guchait, A. Gurtu, G. Kole, S. Kumar, B. Mahakud, M. Maity, G. Majumder, K. Mazumdar, S. Mitra, G. B. Mohanty, B. Parida, T. Sarkar, N. Sur, B. Sutar, N. Wickramage, S. Chauhan, S. Dube, K. Kothekar, S. Sharma, H. Bakhshiansohi, H. Behnamian, S. M. Etesami, A. Fahim, R. Goldouzian, M. Khakzad, M. Mohammadi Najafabadi, M. Naseri, S. Paktinat Mehdiabadi, F. Rezaei Hosseinabadi, B. Safarzadeh, M. Zeinali, M. Felcini, M. Grunewald, M. Abbrescia, C. Calabria, C. Caputo, A. Colaleo, D. Creanza, L. Cristella, N. De Filippis, M. De Palma, L. Fiore, G. Iaselli, G. Maggi, M. Maggi, G. Miniello, S. My, S. Nuzzo, A. Pompili, G. Pugliese, R. Radogna, A. Ranieri, G. Selvaggi, L. Silvestris, R. Venditti, P. Verwilligen, G. Abbiendi, C. Battilana, A. C. Benvenuti, D. Bonacorsi, S. Braibant-Giacomelli, L. Brigliadori, R. Campanini, P. Capiluppi, A. Castro, F. R. Cavallo, S. S. Chhibra, G. Codispoti, M. Cuffiani, G. M. Dallavalle, F. Fabbri, A. Fanfani, D. Fasanella, P. Giacomelli, C. Grandi, L. Guiducci, S. Marcellini, G. Masetti, A. Montanari, F. L. Navarria, A. Perrotta, A. M. Rossi, T. Rovelli, G. P. Siroli, N. Tosi, R. Travaglini, G. Cappello, M. Chiorboli, S. Costa, A. Di Mattia, F. Giordano, R. Potenza, A. Tricomi, C. Tuve, G. Barbagli, V. Ciulli, C. Civinini, R. D’Alessandro, E. Focardi, S. Gonzi, V. Gori, P. Lenzi, M. Meschini, S. Paoletti, G. Sguazzoni, A. Tropiano, L. Viliani, L. Benussi, S. Bianco, F. Fabbri, D. Piccolo, F. Primavera, V. Calvelli, F. Ferro, M. Lo Vetere, M. R. Monge, E. Robutti, S. Tosi, L. Brianza, M. E. Dinardo, S. Fiorendi, S. Gennai, R. Gerosa, A. Ghezzi, P. Govoni, S. Malvezzi, R. A. Manzoni, B. Marzocchi, D. Menasce, L. Moroni, M. Paganoni, D. Pedrini, S. Ragazzi, N. Redaelli, T. Tabarelli de Fatis, S. Buontempo, N. Cavallo, S. Di Guida, M. Esposito, F. Fabozzi, A. O. M. Iorio, G. Lanza, L. Lista, S. Meola, M. Merola, P. Paolucci, C. Sciacca, F. Thyssen, P. Azzi, N. Bacchetta, L. Benato, D. Bisello, A. Boletti, A. Branca, R. Carlin, P. Checchia, M. Dall’Osso, T. Dorigo, U. Dosselli, F. Gasparini, U. Gasparini, A. Gozzelino, K. Kanishchev, S. Lacaprara, M. Margoni, A. T. Meneguzzo, J. Pazzini, N. Pozzobon, P. Ronchese, F. Simonetto, E. Torassa, M. Tosi, S. Ventura, M. Zanetti, P. Zotto, A. Zucchetta, G. Zumerle, A. Braghieri, A. Magnani, P. Montagna, S. P. Ratti, V. Re, C. Riccardi, P. Salvini, I. Vai, P. Vitulo, L. Alunni Solestizi, M. Biasini, G. M. Bilei, D. Ciangottini, L. Fanò, P. Lariccia, G. Mantovani, M. Menichelli, A. Saha, A. Santocchia, K. Androsov, P. Azzurri, G. Bagliesi, J. Bernardini, T. Boccali, R. Castaldi, M. A. Ciocci, R. Dell’Orso, S. Donato, G. Fedi, L. Foà, A. Giassi, M. T. Grippo, F. Ligabue, T. Lomtadze, L. Martini, A. Messineo, F. Palla, A. Rizzi, A. Savoy-Navarro, A. T. Serban, P. Spagnolo, R. Tenchini, G. Tonelli, A. Venturi, P. G. Verdini, L. Barone, F. Cavallari, G. D’imperio, D. Del Re, M. Diemoz, S. Gelli, C. Jorda, E. Longo, F. Margaroli, P. Meridiani, G. Organtini, R. Paramatti, F. Preiato, S. Rahatlou, C. Rovelli, F. Santanastasio, P. Traczyk, N. Amapane, R. Arcidiacono, S. Argiro, M. Arneodo, R. Bellan, C. Biino, N. Cartiglia, M. Costa, R. Covarelli, A. Degano, N. Demaria, L. Finco, B. Kiani, C. Mariotti, S. Maselli, E. Migliore, V. Monaco, E. Monteil, M. M. Obertino, L. Pacher, N. Pastrone, M. Pelliccioni, G. L. Pinna Angioni, F. Ravera, A. Romero, M. Ruspa, R. Sacchi, A. Solano, A. Staiano, U. Tamponi, S. Belforte, V. Candelise, M. Casarsa, F. Cossutti, G. Della Ricca, B. Gobbo, C. La Licata, M. Marone, A. Schizzi, A. Zanetti, A. Kropivnitskaya, S. K. Nam, D. H. Kim, G. N. Kim, M. S. Kim, D. J. Kong, S. Lee, Y. D. Oh, A. Sakharov, D. C. Son, J. A. Brochero Cifuentes, H. Kim, T. J. Kim, S. Song, S. Choi, Y. Go, D. Gyun, B. Hong, M. Jo, H. Kim, Y. Kim, B. Lee, K. Lee, K. S. Lee, S. Lee, S. K. Park, Y. Roh, H. D. Yoo, M. Choi, H. Kim, J. H. Kim, J. S. H. Lee, I. C. Park, G. Ryu, M. S. Ryu, Y. Choi, J. Goh, D. Kim, E. Kwon, J. Lee, I. Yu, V. Dudenas, A. Juodagalvis, J. Vaitkus, I. Ahmed, Z. A. Ibrahim, J. R. Komaragiri, M. A. B. Md Ali, F. Mohamad Idris, W. A. T. Wan Abdullah, M. N. Yusli, E. Casimiro Linares, H. Castilla-Valdez, E. De La Cruz-Burelo, I. Heredia-De La Cruz, A. Hernandez-Almada, R. Lopez-Fernandez, A. Sanchez-Hernandez, S. Carrillo Moreno, F. Vazquez Valencia, I. Pedraza, H. A. Salazar Ibarguen, A. Morelos Pineda, D. Krofcheck, P. H. Butler, A. Ahmad, M. Ahmad, Q. Hassan, H. R. Hoorani, W. A. Khan, T. Khurshid, M. Shoaib, H. Bialkowska, M. Bluj, B. Boimska, T. Frueboes, M. Górski, M. Kazana, K. Nawrocki, K. Romanowska-Rybinska, M. Szleper, P. Zalewski, G. Brona, K. Bunkowski, A. Byszuk, K. Doroba, A. Kalinowski, M. Konecki, J. Krolikowski, M. Misiura, M. Olszewski, M. Walczak, P. Bargassa, C. Beirão Da Cruz E. Silva, A. Di Francesco, P. Faccioli, P. G. Ferreira Parracho, M. Gallinaro, N. Leonardo, L. Lloret Iglesias, F. Nguyen, J. Rodrigues Antunes, J. Seixas, O. Toldaiev, D. Vadruccio, J. Varela, P. Vischia, S. Afanasiev, P. Bunin, M. Gavrilenko, I. Golutvin, I. Gorbunov, A. Kamenev, V. Karjavin, V. Konoplyanikov, A. Lanev, A. Malakhov, V. Matveev, P. Moisenz, V. Palichik, V. Perelygin, S. Shmatov, S. Shulha, N. Skatchkov, V. Smirnov, A. Zarubin, V. Golovtsov, Y. Ivanov, V. Kim, E. Kuznetsova, P. Levchenko, V. Murzin, V. Oreshkin, I. Smirnov, V. Sulimov, L. Uvarov, S. Vavilov, A. Vorobyev, Yu. Andreev, A. Dermenev, S. Gninenko, N. Golubev, A. Karneyeu, M. Kirsanov, N. Krasnikov, A. Pashenkov, D. Tlisov, A. Toropin, V. Epshteyn, V. Gavrilov, N. Lychkovskaya, V. Popov, I. Pozdnyakov, G. Safronov, A. Spiridonov, E. Vlasov, A. Zhokin, A. Bylinkin, V. Andreev, M. Azarkin, I. Dremin, M. Kirakosyan, A. Leonidov, G. Mesyats, S. V. Rusakov, A. Baskakov, A. Belyaev, E. Boos, V. Bunichev, M. Dubinin, L. Dudko, A. Ershov, A. Gribushin, V. Klyukhin, N. Korneeva, I. Lokhtin, I. Myagkov, S. Obraztsov, M. Perfilov, V. Savrin, I. Azhgirey, I. Bayshev, S. Bitioukov, V. Kachanov, A. Kalinin, D. Konstantinov, V. Krychkine, V. Petrov, R. Ryutin, A. Sobol, L. Tourtchanovitch, S. Troshin, N. Tyurin, A. Uzunian, A. Volkov, P. Adzic, J. Milosevic, V. Rekovic, J. Alcaraz Maestre, E. Calvo, M. Cerrada, M. Chamizo Llatas, N. Colino, B. De La Cruz, A. Delgado Peris, D. Domínguez Vázquez, A. Escalante Del Valle, C. Fernandez Bedoya, J. P. Fernández Ramos, J. Flix, M. C. Fouz, P. Garcia-Abia, O. Gonzalez Lopez, S. Goy Lopez, J. M. Hernandez, M. I. Josa, E. Navarro De Martino, A. Pérez-Calero Yzquierdo, J. Puerta Pelayo, A. Quintario Olmeda, I. Redondo, L. Romero, J. Santaolalla, M. S. Soares, C. Albajar, J. F. de Trocóniz, M. Missiroli, D. Moran, J. Cuevas, J. Fernandez Menendez, S. Folgueras, I. Gonzalez Caballero, E. Palencia Cortezon, J. M. Vizan Garcia, I. J. Cabrillo, A. Calderon, J. R. Castiñeiras De Saa, P. De Castro Manzano, J. Duarte Campderros, M. Fernandez, J. Garcia-Ferrero, G. Gomez, A. Lopez Virto, J. Marco, R. Marco, C. Martinez Rivero, F. Matorras, F. J. Munoz Sanchez, J. Piedra Gomez, T. Rodrigo, A. Y. Rodríguez-Marrero, A. Ruiz-Jimeno, L. Scodellaro, N. Trevisani, I. Vila, R. Vilar Cortabitarte, D. Abbaneo, E. Auffray, G. Auzinger, M. Bachtis, P. Baillon, A. H. Ball, D. Barney, A. Benaglia, J. Bendavid, L. Benhabib, J. F. Benitez, G. M. Berruti, P. Bloch, A. Bocci, A. Bonato, C. Botta, H. Breuker, T. Camporesi, R. Castello, G. Cerminara, M. D’Alfonso, D. d’Enterria, A. Dabrowski, V. Daponte, A. David, M. De Gruttola, F. De Guio, A. De Roeck, S. De Visscher, E. Di Marco, M. Dobson, M. Dordevic, B. Dorney, T. du Pree, M. Dünser, N. Dupont, A. Elliott-Peisert, G. Franzoni, W. Funk, D. Gigi, K. Gill, D. Giordano, M. Girone, F. Glege, R. Guida, S. Gundacker, M. Guthoff, J. Hammer, P. Harris, J. Hegeman, V. Innocente, P. Janot, H. Kirschenmann, M. J. Kortelainen, K. Kousouris, K. Krajczar, P. Lecoq, C. Lourenço, M. T. Lucchini, N. Magini, L. Malgeri, M. Mannelli, A. Martelli, L. Masetti, F. Meijers, S. Mersi, E. Meschi, F. Moortgat, S. Morovic, M. Mulders, M. V. Nemallapudi, H. Neugebauer, S. Orfanelli, L. Orsini, L. Pape, E. Perez, M. Peruzzi, A. Petrilli, G. Petrucciani, A. Pfeiffer, D. Piparo, A. Racz, G. Rolandi, M. Rovere, M. Ruan, H. Sakulin, C. Schäfer, C. Schwick, M. Seidel, A. Sharma, P. Silva, M. Simon, P. Sphicas, J. Steggemann, B. Stieger, M. Stoye, Y. Takahashi, D. Treille, A. Triossi, A. Tsirou, G. I. Veres, N. Wardle, H. K. Wöhri, A. Zagozdzinska, W. D. Zeuner, W. Bertl, K. Deiters, W. Erdmann, R. Horisberger, Q. Ingram, H. C. Kaestli, D. Kotlinski, U. Langenegger, D. Renker, T. Rohe, F. Bachmair, L. Bäni, L. Bianchini, B. Casal, G. Dissertori, M. Dittmar, M. Donegà, P. Eller, C. Grab, C. Heidegger, D. Hits, J. Hoss, G. Kasieczka, W. Lustermann, B. Mangano, M. Marionneau, P. Martinez Ruiz del Arbol, M. Masciovecchio, D. Meister, F. Micheli, P. Musella, F. Nessi-Tedaldi, F. Pandolfi, J. Pata, F. Pauss, L. Perrozzi, M. Quittnat, M. Rossini, A. Starodumov, M. Takahashi, V. R. Tavolaro, K. Theofilatos, R. Wallny, T. K. Aarrestad, C. Amsler, L. Caminada, M. F. Canelli, V. Chiochia, A. De Cosa, C. Galloni, A. Hinzmann, T. Hreus, B. Kilminster, C. Lange, J. Ngadiuba, D. Pinna, P. Robmann, F. J. Ronga, D. Salerno, Y. Yang, M. Cardaci, K. H. Chen, T. H. Doan, Sh. Jain, R. Khurana, M. Konyushikhin, C. M. Kuo, W. Lin, Y. J. Lu, S. S. Yu, Arun Kumar, R. Bartek, P. Chang, Y. H. Chang, Y. W. Chang, Y. Chao, K. F. Chen, P. H. Chen, C. Dietz, F. Fiori, U. Grundler, W.-S. Hou, Y. Hsiung, Y. F. Liu, R.-S. Lu, M. Miñano Moya, E. Petrakou, J. f. Tsai, Y. M. Tzeng, B. Asavapibhop, K. Kovitanggoon, G. Singh, N. Srimanobhas, N. Suwonjandee, A. Adiguzel, S. Cerci, Z. S. Demiroglu, C. Dozen, I. Dumanoglu, S. Girgis, G. Gokbulut, Y. Guler, E. Gurpinar, I. Hos, E. E. Kangal, A. Kayis Topaksu, G. Onengut, K. Ozdemir, S. Ozturk, B. Tali, H. Topakli, M. Vergili, C. Zorbilmez, I. V. Akin, B. Bilin, S. Bilmis, B. Isildak, G. Karapinar, M. Yalvac, M. Zeyrek, E. Gülmez, M. Kaya, O. Kaya, E. A. Yetkin, T. Yetkin, A. Cakir, K. Cankocak, S. Sen, F. I. Vardarlı, B. Grynyov, L. Levchuk, P. Sorokin, R. Aggleton, F. Ball, L. Beck, J. J. Brooke, E. Clement, D. Cussans, H. Flacher, J. Goldstein, M. Grimes, G. P. Heath, H. F. Heath, J. Jacob, L. Kreczko, C. Lucas, Z. Meng, D. M. Newbold, S. Paramesvaran, A. Poll, T. Sakuma, S. Seif El Nasr-storey, S. Senkin, D. Smith, V. J. Smith, K. W. Bell, A. Belyaev, C. Brew, R. M. Brown, L. Calligaris, D. Cieri, D. J. A. Cockerill, J. A. Coughlan, K. Harder, S. Harper, E. Olaiya, D. Petyt, C. H. Shepherd-Themistocleous, A. Thea, I. R. Tomalin, T. Williams, W. J. Womersley, S. D. Worm, M. Baber, R. Bainbridge, O. Buchmuller, A. Bundock, D. Burton, S. Casasso, M. Citron, D. Colling, L. Corpe, N. Cripps, P. Dauncey, G. Davies, A. De Wit, M. Della Negra, P. Dunne, A. Elwood, W. Ferguson, J. Fulcher, D. Futyan, G. Hall, G. Iles, M. Kenzie, R. Lane, R. Lucas, L. Lyons, A.-M. Magnan, S. Malik, J. Nash, A. Nikitenko, J. Pela, M. Pesaresi, K. Petridis, D. M. Raymond, A. Richards, A. Rose, C. Seez, A. Tapper, K. Uchida, M. Vazquez Acosta, T. Virdee, S. C. Zenz, J. E. Cole, P. R. Hobson, A. Khan, P. Kyberd, D. Leggat, D. Leslie, I. D. Reid, P. Symonds, L. Teodorescu, M. Turner, A. Borzou, K. Call, J. Dittmann, K. Hatakeyama, H. Liu, N. Pastika, O. Charaf, S. I. Cooper, C. Henderson, P. Rumerio, D. Arcaro, A. Avetisyan, T. Bose, C. Fantasia, D. Gastler, P. Lawson, D. Rankin, C. Richardson, J. Rohlf, J. St. John, L. Sulak, D. Zou, J. Alimena, E. Berry, S. Bhattacharya, D. Cutts, N. Dhingra, A. Ferapontov, A. Garabedian, J. Hakala, U. Heintz, E. Laird, G. Landsberg, Z. Mao, M. Narain, S. Piperov, S. Sagir, R. Syarif, R. Breedon, G. Breto, M. Calderon De La Barca Sanchez, S. Chauhan, M. Chertok, J. Conway, R. Conway, P. T. Cox, R. Erbacher, M. Gardner, W. Ko, R. Lander, M. Mulhearn, D. Pellett, J. Pilot, F. Ricci-Tam, S. Shalhout, J. Smith, M. Squires, D. Stolp, M. Tripathi, S. Wilbur, R. Yohay, R. Cousins, P. Everaerts, C. Farrell, J. Hauser, M. Ignatenko, D. Saltzberg, E. Takasugi, V. Valuev, M. Weber, K. Burt, R. Clare, J. Ellison, J. W. Gary, G. Hanson, J. Heilman, M. Ivova Paneva, P. Jandir, E. Kennedy, F. Lacroix, O. R. Long, A. Luthra, M. Malberti, M. Olmedo Negrete, A. Shrinivas, H. Wei, S. Wimpenny, B. R. Yates, J. G. Branson, G. B. Cerati, S. Cittolin, R. T. D’Agnolo, M. Derdzinski, A. Holzner, R. Kelley, D. Klein, J. Letts, I. Macneill, D. Olivito, S. Padhi, M. Pieri, M. Sani, V. Sharma, S. Simon, M. Tadel, A. Vartak, S. Wasserbaech, C. Welke, F. Würthwein, A. Yagil, G. Zevi Della Porta, J. Bradmiller-Feld, C. Campagnari, A. Dishaw, V. Dutta, K. Flowers, M. Franco Sevilla, P. Geffert, C. George, F. Golf, L. Gouskos, J. Gran, J. Incandela, N. Mccoll, S. D. Mullin, J. Richman, D. Stuart, I. Suarez, C. West, J. Yoo, D. Anderson, A. Apresyan, A. Bornheim, J. Bunn, Y. Chen, J. Duarte, A. Mott, H. B. Newman, C. Pena, M. Pierini, M. Spiropulu, J. R. Vlimant, S. Xie, R. Y. Zhu, M. B. Andrews, V. Azzolini, A. Calamba, B. Carlson, T. Ferguson, M. Paulini, J. Russ, M. Sun, H. Vogel, I. Vorobiev, J. P. Cumalat, W. T. Ford, A. Gaz, F. Jensen, A. Johnson, M. Krohn, T. Mulholland, U. Nauenberg, K. Stenson, S. R. Wagner, J. Alexander, A. Chatterjee, J. Chaves, J. Chu, S. Dittmer, N. Eggert, N. Mirman, G. Nicolas Kaufman, J. R. Patterson, A. Rinkevicius, A. Ryd, L. Skinnari, L. Soffi, W. Sun, S. M. Tan, W. D. Teo, J. Thom, J. Thompson, J. Tucker, Y. Weng, P. Wittich, S. Abdullin, M. Albrow, J. Anderson, G. Apollinari, S. Banerjee, L. A. T. Bauerdick, A. Beretvas, J. Berryhill, P. C. Bhat, G. Bolla, K. Burkett, J. N. Butler, H. W. K. Cheung, F. Chlebana, S. Cihangir, V. D. Elvira, I. Fisk, J. Freeman, E. Gottschalk, L. Gray, D. Green, S. Grünendahl, O. Gutsche, J. Hanlon, D. Hare, R. M. Harris, S. Hasegawa, J. Hirschauer, Z. Hu, B. Jayatilaka, S. Jindariani, M. Johnson, U. Joshi, A. W. Jung, B. Klima, B. Kreis, S. Kwan, S. Lammel, J. Linacre, D. Lincoln, R. Lipton, T. Liu, R. Lopes De Sá, J. Lykken, K. Maeshima, J. M. Marraffino, V. I. Martinez Outschoorn, S. Maruyama, D. Mason, P. McBride, P. Merkel, K. Mishra, S. Mrenna, S. Nahn, C. Newman-Holmes, V. O’Dell, K. Pedro, O. Prokofyev, G. Rakness, E. Sexton-Kennedy, A. Soha, W. J. Spalding, L. Spiegel, L. Taylor, S. Tkaczyk, N. V. Tran, L. Uplegger, E. W. Vaandering, C. Vernieri, M. Verzocchi, R. Vidal, H. A. Weber, A. Whitbeck, F. Yang, D. Acosta, P. Avery, P. Bortignon, D. Bourilkov, A. Carnes, M. Carver, D. Curry, S. Das, G. P. Di Giovanni, R. D. Field, I. K. Furic, S. V. Gleyzer, J. Hugon, J. Konigsberg, A. Korytov, J. F. Low, P. Ma, K. Matchev, H. Mei, P. Milenovic, G. Mitselmakher, D. Rank, R. Rossin, L. Shchutska, M. Snowball, D. Sperka, N. Terentyev, L. Thomas, J. Wang, S. Wang, J. Yelton, S. Hewamanage, S. Linn, P. Markowitz, G. Martinez, J. L. Rodriguez, A. Ackert, J. R. Adams, T. Adams, A. Askew, J. Bochenek, B. Diamond, J. Haas, S. Hagopian, V. Hagopian, K. F. Johnson, A. Khatiwada, H. Prosper, M. Weinberg, M. M. Baarmand, V. Bhopatkar, S. Colafranceschi, M. Hohlmann, H. Kalakhety, D. Noonan, T. Roy, F. Yumiceva, M. R. Adams, L. Apanasevich, D. Berry, R. R. Betts, I. Bucinskaite, R. Cavanaugh, O. Evdokimov, L. Gauthier, C. E. Gerber, D. J. Hofman, P. Kurt, C. O’Brien, I. D. Sandoval Gonzalez, C. Silkworth, P. Turner, N. Varelas, Z. Wu, M. Zakaria, B. Bilki, W. Clarida, K. Dilsiz, S. Durgut, R. P. Gandrajula, M. Haytmyradov, V. Khristenko, J.-P. Merlo, H. Mermerkaya, A. Mestvirishvili, A. Moeller, J. Nachtman, H. Ogul, Y. Onel, F. Ozok, A. Penzo, C. Snyder, E. Tiras, J. Wetzel, K. Yi, I. Anderson, B. A. Barnett, B. Blumenfeld, N. Eminizer, D. Fehling, L. Feng, A. V. Gritsan, P. Maksimovic, C. Martin, M. Osherson, J. Roskes, A. Sady, U. Sarica, M. Swartz, M. Xiao, Y. Xin, C. You, P. Baringer, A. Bean, G. Benelli, C. Bruner, R. P. Kenny, D. Majumder, M. Malek, M. Murray, S. Sanders, R. Stringer, Q. Wang, A. Ivanov, K. Kaadze, S. Khalil, M. Makouski, Y. Maravin, A. Mohammadi, L. K. Saini, N. Skhirtladze, S. Toda, D. Lange, F. Rebassoo, D. Wright, C. Anelli, A. Baden, O. Baron, A. Belloni, B. Calvert, S. C. Eno, C. Ferraioli, J. A. Gomez, N. J. Hadley, S. Jabeen, R. G. Kellogg, T. Kolberg, J. Kunkle, Y. Lu, A. C. Mignerey, Y. H. Shin, A. Skuja, M. B. Tonjes, S. C. Tonwar, A. Apyan, R. Barbieri, A. Baty, K. Bierwagen, S. Brandt, W. Busza, I. A. Cali, Z. Demiragli, L. Di Matteo, G. Gomez Ceballos, M. Goncharov, D. Gulhan, Y. Iiyama, G. M. Innocenti, M. Klute, D. Kovalskyi, Y. S. Lai, Y.-J. Lee, A. Levin, P. D. Luckey, A. C. Marini, C. Mcginn, C. Mironov, S. Narayanan, X. Niu, C. Paus, D. Ralph, C. Roland, G. Roland, J. Salfeld-Nebgen, G. S. F. Stephans, K. Sumorok, M. Varma, D. Velicanu, J. Veverka, J. Wang, T. W. Wang, B. Wyslouch, M. Yang, V. Zhukova, B. Dahmes, A. Evans, A. Finkel, A. Gude, P. Hansen, S. Kalafut, S. C. Kao, K. Klapoetke, Y. Kubota, Z. Lesko, J. Mans, S. Nourbakhsh, N. Ruckstuhl, R. Rusack, N. Tambe, J. Turkewitz, J. G. Acosta, S. Oliveros, E. Avdeeva, K. Bloom, S. Bose, D. R. Claes, A. Dominguez, C. Fangmeier, R. Gonzalez Suarez, R. Kamalieddin, J. Keller, D. Knowlton, I. Kravchenko, F. Meier, J. Monroy, F. Ratnikov, J. E. Siado, G. R. Snow, M. Alyari, J. Dolen, J. George, A. Godshalk, C. Harrington, I. Iashvili, J. Kaisen, A. Kharchilava, A. Kumar, S. Rappoccio, B. Roozbahani, G. Alverson, E. Barberis, D. Baumgartel, M. Chasco, A. Hortiangtham, A. Massironi, D. M. Morse, D. Nash, T. Orimoto, R. Teixeira De Lima, D. Trocino, R.-J. Wang, D. Wood, J. Zhang, K. A. Hahn, A. Kubik, N. Mucia, N. Odell, B. Pollack, A. Pozdnyakov, M. Schmitt, S. Stoynev, K. Sung, M. Trovato, M. Velasco, A. Brinkerhoff, N. Dev, M. Hildreth, C. Jessop, D. J. Karmgard, N. Kellams, K. Lannon, S. Lynch, N. Marinelli, F. Meng, C. Mueller, Y. Musienko, T. Pearson, M. Planer, A. Reinsvold, R. Ruchti, G. Smith, S. Taroni, N. Valls, M. Wayne, M. Wolf, A. Woodard, L. Antonelli, J. Brinson, B. Bylsma, L. S. Durkin, S. Flowers, A. Hart, C. Hill, R. Hughes, W. Ji, K. Kotov, T. Y. Ling, B. Liu, W. Luo, D. Puigh, M. Rodenburg, B. L. Winer, H. W. Wulsin, O. Driga, P. Elmer, J. Hardenbrook, P. Hebda, S. A. Koay, P. Lujan, D. Marlow, T. Medvedeva, M. Mooney, J. Olsen, C. Palmer, P. Piroué, H. Saka, D. Stickland, C. Tully, A. Zuranski, S. Malik, V. E. Barnes, D. Benedetti, D. Bortoletto, L. Gutay, M. K. Jha, M. Jones, K. Jung, D. H. Miller, N. Neumeister, B. C. Radburn-Smith, X. Shi, I. Shipsey, D. Silvers, J. Sun, A. Svyatkovskiy, F. Wang, W. Xie, L. Xu, N. Parashar, J. Stupak, A. Adair, B. Akgun, Z. Chen, K. M. Ecklund, F. J. M. Geurts, M. Guilbaud, W. Li, B. Michlin, M. Northup, B. P. Padley, R. Redjimi, J. Roberts, J. Rorie, Z. Tu, J. Zabel, B. Betchart, A. Bodek, P. de Barbaro, R. Demina, Y. Eshaq, T. Ferbel, M. Galanti, A. Garcia-Bellido, J. Han, A. Harel, O. Hindrichs, A. Khukhunaishvili, G. Petrillo, P. Tan, M. Verzetti, S. Arora, A. Barker, J. P. Chou, C. Contreras-Campana, E. Contreras-Campana, D. Duggan, D. Ferencek, Y. Gershtein, R. Gray, E. Halkiadakis, D. Hidas, E. Hughes, S. Kaplan, R. Kunnawalkam Elayavalli, A. Lath, K. Nash, S. Panwalkar, M. Park, S. Salur, S. Schnetzer, D. Sheffield, S. Somalwar, R. Stone, S. Thomas, P. Thomassen, M. Walker, M. Foerster, G. Riley, K. Rose, S. Spanier, A. York, O. Bouhali, A. Castaneda Hernandez, M. Dalchenko, M. De Mattia, A. Delgado, S. Dildick, R. Eusebi, J. Gilmore, T. Kamon, V. Krutelyov, R. Mueller, I. Osipenkov, Y. Pakhotin, R. Patel, A. Perloff, A. Rose, A. Safonov, A. Tatarinov, K. A. Ulmer, N. Akchurin, C. Cowden, J. Damgov, C. Dragoiu, P. R. Dudero, J. Faulkner, S. Kunori, K. Lamichhane, S. W. Lee, T. Libeiro, S. Undleeb, I. Volobouev, E. Appelt, A. G. Delannoy, S. Greene, A. Gurrola, R. Janjam, W. Johns, C. Maguire, Y. Mao, A. Melo, H. Ni, P. Sheldon, B. Snook, S. Tuo, J. Velkovska, Q. Xu, M. W. Arenton, B. Cox, B. Francis, J. Goodell, R. Hirosky, A. Ledovskoy, H. Li, C. Lin, C. Neu, T. Sinthuprasith, X. Sun, Y. Wang, E. Wolfe, J. Wood, F. Xia, C. Clarke, R. Harr, P. E. Karchin, C. Kottachchi Kankanamge Don, P. Lamichhane, J. Sturdy, D. A. Belknap, D. Carlsmith, M. Cepeda, S. Dasu, L. Dodd, S. Duric, B. Gomber, M. Grothe, R. Hall-Wilton, M. Herndon, A. Hervé, P. Klabbers, A. Lanaro, A. Levine, K. Long, R. Loveless, A. Mohapatra, I. Ojalvo, T. Perry, G. A. Pierro, G. Polese, T. Ruggles, T. Sarangi, A. Savin, A. Sharma, N. Smith, W. H. Smith, D. Taylor, N. Woods, ATLAS Collaboration

**Affiliations:** 1 Yerevan Physics Institute, Yerevan, Armenia; 2 Institut für Hochenergiephysik der OeAW, Vienna, Austria; 3 National Centre for Particle and High Energy Physics, Minsk, Belarus; 4 Universiteit Antwerpen, Antwerpen, Belgium; 5 Vrije Universiteit Brussel, Brussel, Belgium; 6 Université Libre de Bruxelles, Bruxelles, Belgium; 7 Ghent University, Ghent, Belgium; 8 Université Catholique de Louvain, Louvain-la-Neuve, Belgium; 9 Université de Mons, Mons, Belgium; 10 Centro Brasileiro de Pesquisas Fisicas, Rio de Janeiro, Brazil; 11 Universidade do Estado do Rio de Janeiro, Rio de Janeiro, Brazil; 12 Universidade Estadual Paulista, Universidade Federal do ABC, São Paulo, Brazil; 13 Institute for Nuclear Research and Nuclear Energy, Sofia, Bulgaria; 14 University of Sofia, Sofia, Bulgaria; 15 Institute of High Energy Physics, Beijing, China; 16 State Key Laboratory of Nuclear Physics and Technology, Peking University, Beijing, China; 17 Universidad de Los Andes, Bogota, Colombia; 18 Faculty of Electrical Engineering, Mechanical Engineering and Naval Architecture, University of Split, Split, Croatia; 19 Faculty of Science, University of Split, Split, Croatia; 20 Institute Rudjer Boskovic, Zagreb, Croatia; 21 University of Cyprus, Nicosia, Cyprus; 22 Charles University, Prague, Czech Republic; 23 Academy of Scientific Research and Technology of the Arab Republic of Egypt, Egyptian Network of High Energy Physics, Cairo, Egypt; 24 National Institute of Chemical Physics and Biophysics, Tallinn, Estonia; 25 Department of Physics, University of Helsinki, Helsinki, Finland; 26 Helsinki Institute of Physics, Helsinki, Finland; 27 Lappeenranta University of Technology, Lappeenranta, Finland; 28 DSM/IRFU, CEA/Saclay, Gif-sur-Yvette, France; 29 Laboratoire Leprince-Ringuet, Ecole Polytechnique, IN2P3-CNRS, Palaiseau, France; 30 Institut Pluridisciplinaire Hubert Curien, Université de Strasbourg, Université de Haute Alsace Mulhouse, CNRS/IN2P3, Strasbourg, France; 31 Centre de Calcul de l’Institut National de Physique Nucleaire et de Physique des Particules, CNRS/IN2P3, Villeurbanne, France; 32 Université de Lyon, Université Claude Bernard Lyon 1, CNRS-IN2P3, Institut de Physique Nucléaire de Lyon, Villeurbanne, France; 33 Georgian Technical University, Tbilisi, Georgia; 34 Tbilisi State University, Tbilisi, Georgia; 35 I. Physikalisches Institut, RWTH Aachen University, Aachen, Germany; 36 III. Physikalisches Institut A, RWTH Aachen University, Aachen, Germany; 37 III. Physikalisches Institut B, RWTH Aachen University, Aachen, Germany; 38 Deutsches Elektronen-Synchrotron, Hamburg, Germany; 39 University of Hamburg, Hamburg, Germany; 40 Institut für Experimentelle Kernphysik, Karlsruhe, Germany; 41 Institute of Nuclear and Particle Physics (INPP), NCSR Demokritos, Aghia Paraskevi, Greece; 42 National and Kapodistrian University of Athens, Athens, Greece; 43 University of Ioánnina, Ioánnina, Greece; 44 Wigner Research Centre for Physics, Budapest, Hungary; 45 Institute of Nuclear Research ATOMKI, Debrecen, Hungary; 46 University of Debrecen, Debrecen, Hungary; 47 National Institute of Science Education and Research, Bhubaneswar, India; 48 Panjab University, Chandigarh, India; 49 University of Delhi, Delhi, India; 50 Saha Institute of Nuclear Physics, Kolkata, India; 51 Bhabha Atomic Research Centre, Mumbai, India; 52 Tata Institute of Fundamental Research, Mumbai, India; 53 Indian Institute of Science Education and Research (IISER), Pune, India; 54 Institute for Research in Fundamental Sciences (IPM), Tehran, Iran; 55 University College Dublin, Dublin, Ireland; 56 INFN Sezione di Bari, Università di Bari, Politecnico di Bari, Bari, Italy; 57 INFN Sezione di Bologna, Università di Bologna, Bologna, Italy; 58 INFN Sezione di Catania, Università di Catania, Catania, Italy; 59 INFN Sezione di Firenze, Università di Firenze, Florence, Italy; 60 INFN Laboratori Nazionali di Frascati, Frascati, Italy; 61 INFN Sezione di Genova, Università di Genova, Genoa, Italy; 62 INFN Sezione di Milano-Bicocca, Università di Milano-Bicocca, Milan, Italy; 63 INFN Sezione di Napoli, Università di Napoli Federico II’, Naples, Italy, Università della Basilicata, Potenza, Italy, Università G. Marconi, Rome, Italy; 64 INFN Sezione di Padova, Università di Padova, Padova, Italy, Università di Trento, Trento, Italy; 65 INFN Sezione di Pavia, Università di Pavia, Pavia, Italy; 66 INFN Sezione di Perugia, Università di Perugia, Perugia, Italy; 67 INFN Sezione di Pisa, Università di Pisa, Scuola Normale Superiore di Pisa, Pisa, Italy; 68 INFN Sezione di Roma, Università di Roma, Rome, Italy; 69 INFN Sezione di Torino, Università di Torino, Turin, Italy, Università del Piemonte Orientale, Novara, Italy; 70 INFN Sezione di Trieste, Università di Trieste, Trieste, Italy; 71 Kangwon National University, Chunchon, Korea; 72 Kyungpook National University, Daegu, Korea; 73 Chonbuk National University, Jeonju, Korea; 74 Chonnam National University, Institute for Universe and Elementary Particles, Kwangju, Korea; 75 Korea University, Seoul, Korea; 76 Seoul National University, Seoul, Korea; 77 University of Seoul, Seoul, Korea; 78 Sungkyunkwan University, Suwon, Korea; 79 Vilnius University, Vilnius, Lithuania; 80 National Centre for Particle Physics, Universiti Malaya, Kuala Lumpur, Malaysia; 81 Centro de Investigacion y de Estudios Avanzados del IPN, Mexico City, Mexico; 82 Universidad Iberoamericana, Mexico City, Mexico; 83 Benemerita Universidad Autonoma de Puebla, Puebla, Mexico; 84 Universidad Autónoma de San Luis Potosí, San Luis Potosí, Mexico; 85 University of Auckland, Auckland, New Zealand; 86 University of Canterbury, Christchurch, New Zealand; 87 National Centre for Physics, Quaid-I-Azam University, Islamabad, Pakistan; 88 National Centre for Nuclear Research, Swierk, Poland; 89 Faculty of Physics, Institute of Experimental Physics University of Warsaw, Warsaw, Poland; 90 Laboratório de Instrumentação e Física Experimental de Partículas, Lisboa, Portugal; 91 Joint Institute for Nuclear Research, Dubna, Russia; 92 Petersburg Nuclear Physics Institute, Gatchina (St. Petersburg), Russia; 93 Institute for Nuclear Research, Moscow, Russia; 94 Institute for Theoretical and Experimental Physics, Moscow, Russia; 95 National Research Nuclear University ‘Moscow Engineering Physics Institute’ (MEPhI), Moscow, Russia; 96 P.N. Lebedev Physical Institute, Moscow, Russia; 97 Skobeltsyn Institute of Nuclear Physics, Lomonosov Moscow State University, Moscow, Russia; 98 State Research Center of Russian Federation, Institute for High Energy Physics, Protvino, Russia; 99 Faculty of Physics and Vinca Institute of Nuclear Sciences, University of Belgrade, Belgrade, Serbia; 100 Centro de Investigaciones Energéticas Medioambientales y Tecnológicas (CIEMAT), Madrid, Spain; 101 Universidad Autónoma de Madrid, Madrid, Spain; 102 Universidad de Oviedo, Oviedo, Spain; 103 Instituto de Física de Cantabria (IFCA), CSIC-Universidad de Cantabria, Santander, Spain; 104 CERN, European Organization for Nuclear Research, Geneva, Switzerland; 105 Paul Scherrer Institut, Villigen, Switzerland; 106 Institute for Particle Physics, ETH Zurich, Zurich, Switzerland; 107 Universität Zürich, Zurich, Switzerland; 108 National Central University, Chung-Li, Taiwan; 109 National Taiwan University (NTU), Taipei, Taiwan; 110 Department of Physics, Faculty of Science, Chulalongkorn University, Bangkok, Thailand; 111 Cukurova University, Adana, Turkey; 112 Physics Department, Middle East Technical University, Ankara, Turkey; 113 Bogazici University, Istanbul, Turkey; 114 Istanbul Technical University, Istanbul, Turkey; 115 Institute for Scintillation Materials of National Academy of Science of Ukraine, Kharkov, Ukraine; 116 National Scientific Center, Kharkov Institute of Physics and Technology, Kharkov, Ukraine; 117 University of Bristol, Bristol, UK; 118 Rutherford Appleton Laboratory, Didcot, UK; 119 Imperial College, London, UK; 120 Brunel University, Uxbridge, UK; 121 Baylor University, Waco, USA; 122 The University of Alabama, Tuscaloosa, USA; 123 Boston University, Boston, USA; 124 Brown University, Providence, USA; 125 University of California, Davis, Davis, USA; 126 University of California, Los Angeles, USA; 127 University of California, Riverside, Riverside, USA; 128 University of California, San Diego, La Jolla, USA; 129 University of California, Santa Barbara, Santa Barbara, USA; 130 California Institute of Technology, Pasadena, USA; 131 Carnegie Mellon University, Pittsburgh, USA; 132 University of Colorado Boulder, Boulder, USA; 133 Cornell University, Ithaca, USA; 134 Fermi National Accelerator Laboratory, Batavia, USA; 135 University of Florida, Gainesville, USA; 136 Florida International University, Miami, USA; 137 Florida State University, Tallahassee, USA; 138 Florida Institute of Technology, Melbourne, USA; 139 University of Illinois at Chicago (UIC), Chicago, USA; 140 The University of Iowa, Iowa City, USA; 141 Johns Hopkins University, Baltimore, USA; 142 The University of Kansas, Lawrence, USA; 143 Kansas State University, Manhattan, USA; 144 Lawrence Livermore National Laboratory, Livermore, USA; 145 University of Maryland, College Park, USA; 146 Massachusetts Institute of Technology, Cambridge, USA; 147 University of Minnesota, Minneapolis, USA; 148 University of Mississippi, Oxford, USA; 149 University of Nebraska-Lincoln, Lincoln, USA; 150 State University of New York at Buffalo, Buffalo, USA; 151 Northeastern University, Boston, USA; 152 Northwestern University, Evanston, USA; 153 University of Notre Dame, Notre Dame, USA; 154 The Ohio State University, Columbus, USA; 155 Princeton University, Princeton, USA; 156 University of Puerto Rico, Mayaguez, USA; 157 Purdue University, West Lafayette, USA; 158 Purdue University Calumet, Hammond, USA; 159 Rice University, Houston, USA; 160 University of Rochester, Rochester, USA; 161 Rutgers, The State University of New Jersey, Piscataway, USA; 162 University of Tennessee, Knoxville, USA; 163 Texas A&M University, College Station, USA; 164 Texas Tech University, Lubbock, USA; 165 Vanderbilt University, Nashville, USA; 166 University of Virginia, Charlottesville, USA; 167 Wayne State University, Detroit, USA; 168 University of Wisconsin, Madison, Madison, WI USA; 169 CERN, 1211 Geneva 23, Switzerland

## Abstract

A measurement of the top quark pair production ($\mathrm{t}\overline{\mathrm{t}}$) cross section in proton–proton collisions at the centre-of-mass energy of 8$\,\text {TeV}$ is presented using data collected with the CMS detector at the LHC, corresponding to an integrated luminosity of 19.6$\,\text {fb}^{-\text {1}}$. This analysis is performed in the $\mathrm{t}\overline{\mathrm{t}}$ decay channels with one isolated, high transverse momentum electron or muon and at least four jets, at least one of which is required to be identified as originating from hadronization of a b quark. The calibration of the jet energy scale and the efficiency of b jet identification are determined from data. The measured $\mathrm{t}\overline{\mathrm{t}}$ cross section is $228.5 \pm 3.8\,\text {(stat)} \pm 13.7\,\text {(syst)} \pm 6.0\,\text {(lumi)} \text { pb}$. This measurement is compared with an analysis of 7$\,\text {TeV}$ data, corresponding to an integrated luminosity of 5.0$\,\text {fb}^{-\text {1}}$, to determine the ratio of 8$\,\text {TeV}$ to 7$\,\text {TeV}$ cross sections, which is found to be $1.43 \pm 0.04\,\text {(stat)} \pm 0.07\,\text {(syst)} \pm 0.05\,\text {(lumi)}$. The measurements are in agreement with QCD predictions up to next-to-next-to-leading order.

## Introduction

Top quarks are abundantly produced at the CERN LHC. The predicted top quark pair production cross section ($\sigma _{\mathrm{t}\overline{\mathrm{t}}}$) in proton–proton (pp) collisions, at a centre-of-mass energy of $8\,\text {TeV}$, is 253$\text { pb}$, with theoretical uncertainties at the level of 5–6%. A precise measurement of $\sigma _{\mathrm{t}\overline{\mathrm{t}}}$ is an important test of perturbative quantum chromodynamics (QCD) at high energies. Furthermore, precision $\mathrm{t}\overline{\mathrm{t}}$ cross section measurements can be used to constrain the top quark mass $m_{\mathrm{t}}$ and QCD parameters, such as the strong coupling constant $\alpha _S$ [1], or the parton distribution functions (PDF) of the proton [2].

The $\mathrm{t}\overline{\mathrm{t}}$ production cross section was measured at the LHC at $\sqrt{s} = 7$ and 8$\,\text {TeV}$  [3–18, 18–25]. In this paper, a measurement of the $\mathrm{t}\overline{\mathrm{t}}$ production cross section in the final state with one high transverse momentum lepton (muon or electron) and jets is presented using the 2012 data set at $\sqrt{s} = 8\,\text {TeV}$, collected by the CMS experiment at the LHC and corresponding to an integrated luminosity of 19.6$\,\text {fb}^{-\text {1}}$. To measure the cross section ratio, where several systematic uncertainties cancel, the 2011 data set at $\sqrt{s} = 7\,\text {TeV}$, corresponding to an integrated luminosity of 5.0$\,\text {fb}^{-\text {1}}$, has been concurrently analyzed with a similar strategy to the one developed for the cross section measurement at 8$\,\text {TeV}$. The new measurement agrees very well with the previously published CMS result [8]. The larger statistical uncertainty of the present measurement with respect to the previous one is due to the simultaneous determination of the $\mathrm{b}$ tagging efficiency, as discussed in Sect. 6. Similarly to the 8$\,\text {TeV}$ analysis, an additional signal modelling uncertainty has been considered in the 7$\,\text {TeV}$ analysis, as reported in Sect. 6.

In the standard model, top quarks are predominantly produced in pairs via the strong interaction and decay almost exclusively into a $\mathrm {W}$ boson and a $\mathrm{b}$ quark. The event signature is determined by the subsequent decays of the two $\mathrm {W}$ bosons. This analysis uses lepton+jets decays into muons or electrons, where one of the $\mathrm {W}$ bosons decays into two quarks and the other to a lepton and a neutrino. Decays of the $\mathrm {W}$ boson into a tau lepton and a neutrino can enter the selection if the tau lepton decays leptonically. The top quark decaying into a $\mathrm{b}$ quark and a leptonically decaying $\mathrm {W}$ boson is defined in the following as the “leptonic top quark”, while the other top quark is referred to as “hadronic top quark”. For the $\mathrm{t}\overline{\mathrm{t}}$ signal two jets result from the hadronization of the $\mathrm{b}$ and $\overline{\mathrm{b}}$ quarks ($\mathrm{b}$ jets), thus $\mathrm{b}$ tagging algorithms are employed for the identification of $\mathrm{b}$ jets in order to improve the purity of the $\mathrm{t}\overline{\mathrm{t}}$ candidate sample.

The technique for extracting the $\mathrm{t}\overline{\mathrm{t}}$ cross section consists of a binned log-likelihood fit of signal and background to the distribution of a discriminant variable in data showing a good separation between signal and background: the invariant mass of the $\mathrm{b}$ jet related to the leptonic top quark and the lepton $\ell$ ($M_{\ell \mathrm{b}}$). The mass of the three-jet combination with the highest transverse momentum in the event ($M_3$) is used as a discriminant in an alternative analysis. The $M_{\ell \mathrm{b}}$ variable is related to the leptonic top quark mass, while $M_3$ is a measure for the hadronic top quark mass. Both quantities provide a good separation between signal and background processes.

The analysis employs calibration techniques to reduce the experimental uncertainties related to $\mathrm{b}$ tagging efficiencies and jet energy scale (JES). The $\mathrm{t}\overline{\mathrm{t}}$ topology is reconstructed using a jet sorting algorithm in which the $\mathrm{b}$ jet most likely originating from the leptonic top quark is identified. The $\mathrm{b}$ tagging efficiency is then determined from a $\mathrm{b}$-enriched sample, in the peak region of the $M_{\ell \mathrm{b}}$ distribution, correcting for the contamination from non-$\mathrm{b}$ jets, following the method described in Refs. [26, 27]. The rate of jets that are wrongly tagged as originating from a $\mathrm{b}$ quark is also measured using data as described in [28]. Independently, the JES is determined using the jets associated with the hadronically decaying $\mathrm {W}$ boson by correcting the reconstructed mass of the $\mathrm {W}$ boson in the simulation to that determined from the data.

The results of the cross section measurements are given both for the visible region, i.e. for the phase space corresponding to the event selection, and for the full phase space. The visible region is defined by requiring the presence in the simulation of exactly one lepton, one neutrino, and at least four jets passing the selection criteria, as presented in Sect. 5.

This paper is structured as follows: after a description of the CMS detector (see Sect. 2), the data and the simulated samples are discussed in Sect. 3, while Sect. 4 is dedicated to the event selection. The analysis technique and the impact of the systematic uncertainties are addressed in Sect. 5 and in Sect. 6. The results of the cross section measurements are discussed in Sect. 7. Section 8 describes the alternative analysis based on $M_3$, followed by a summary in Sect. 9.

## The CMS detector

The central feature of the CMS apparatus is a superconducting solenoid, of 6 m internal diameter, providing an axial magnetic field of 3.8$\text { T}$. Within the solenoidal field volume are a silicon pixel and strip tracker which measure charged particle trajectories in the pseudorapidity range $|\eta | < 2.5$. Also within the field volume, the silicon detectors are surrounded by a lead tungstate crystal electromagnetic calorimeter ($|\eta |<3.0$) and a brass and scintillator hadron calorimeter ($|\eta |<5.0$) that provide high-resolution energy and direction measurements of electrons and hadronic jets. Muons are measured in gas-ionization detectors embedded in the steel magnetic flux-return yoke outside the solenoid. The muon detection systems provide muon detection in the range $|\eta | < 2.4$. A two-level trigger system selects the pp collision events for use in physics analysis. A more detailed description of the CMS detector, together with a definition of the coordinate system used and the relevant kinematic variables, can be found elsewhere [29].

## Data and simulation

The cross section measurement is performed using the 8$\,\text {TeV}$ pp collisions recorded by the CMS experiment in 2012, corresponding to an integrated luminosity of $19.6 \pm 0.5$ $\,\text {fb}^{-\text {1}}$ [30], and the 2011 data set at $\sqrt{s} = 7\,\text {TeV}$, corresponding to an integrated luminosity of $5.0 \pm 0.2$ $\,\text {fb}^{-\text {1}}$ [31].

The $\mathrm{t}\overline{\mathrm{t}}$ events are simulated using the Monte Carlo (MC) event generators MadGraph (version 5.1.1.0) [32, 33] and powheg (v1.0 r1380) [34, 35]. In MadGraph the top quark pairs are generated at leading order with up to three additional high-$p_{\mathrm {T}}$ jets. The powheg generator implements matrix elements to next-to-leading order (NLO) in perturbative QCD, with up to one additional jet. The mass of the top quark is set to $172.5\,\text {GeV}$. The CT10 [36] PDF set is used by powheg and the CTEQ6M [37–39] by MadGraph. The pythia (v.6.426) [40] and herwig (v.6.520) [41] generators are used to model the parton showering. The pythia shower matching is done using the MLM prescription [42, 43].

The top quark pair production cross section values are predicted to be $177.3^{+4.6}_{-6.0}~\text {(scale)} \pm 9.0~(\mathrm {PDF}{+}\alpha _S)\text { pb}$ at 7$\,\text {TeV}$ and $252.9^{+6.4}_{-8.6}~\text {(scale)} \pm 11.7~(\mathrm {PDF}{+}\alpha _S)\text { pb}$ at 8$\,\text {TeV}$, as calculated with the Top++ 2.0 program to next-to-next-to-leading order (NNLO) in perturbative QCD, including soft-gluon resummation to next-to-next-to-leading logarithmic (NNLL) order (Ref. [44] and references therein), and assuming $m_{\mathrm{t}} = 172.5\,\text {GeV}$. The first uncertainty comes from the independent variation of the factorization and renormalization scales, while the second one is associated to variations in the PDF and $\alpha _S$ following the PDF4LHC prescription with the MSTW2008 68% confidence level NNLO, CT10 NNLO, and NNPDF2.3 5f FFN PDF sets (Refs. [37, 38] and references therein, and Refs. [36, 39]).

The top quark transverse momentum is reweighted in samples simulated with MadGraph and powheg, when interfaced to pythia, in order to better describe the $p_{\mathrm {T}}$ distribution observed in the data. Based on studies of differential distributions [45, 46] in the top quark transverse momentum, an event weight $w = \sqrt{\smash [b]{w_1 \, w_2}}$ is applied, where the weights $w_i$ of the two top quarks are given as a function of the generated top quark $p_{\mathrm {T}}$ values: $w_i = \exp (0.199 - 0.00166 \ p_{\mathrm {T}} ^i/{\text {GeV}})$ at 7$\,\text {TeV}$, and $w_i = \exp (0.156 - 0.00137 \ p_{\mathrm {T}} ^i/{\text {GeV}})$ at 8$\,\text {TeV}$. This reweighting is only applied to the phase space corresponding to the experimental selections in the muon and electron channels. The agreement between data and samples generated with powheg interfaced with herwig is found to be satisfactory, and no reweighting is applied in this case.

The $\mathrm {W}/\mathrm{Z}$+jets events, i.e. the associated production of $\mathrm {W}/\mathrm{Z}$ vector bosons with jets, with leptonic decays of the $\mathrm {W}/\mathrm{Z}$ bosons, constitute the largest background. These are also simulated using MadGraph with matrix elements corresponding to at least one jet and up to four jets. The $\mathrm {W}/\mathrm{Z}$+jets events are generated inclusively with respect to the jet flavour. Drell–Yan production of charged leptons is generated for dilepton invariant masses above 50$\,\text {GeV}$, as those events constitute the relevant background in the phase space of this analysis. The contribution from Drell–Yan events with dilepton invariant masses below 50$\,\text {GeV}$ is negligible, as verified with a sample with a mass range of 10–50$\,\text {GeV}$. Single top quark production is simulated with powheg. The background processes are normalized to NLO and NNLO cross section calculations [47–51], with the exception of the QCD multijet background, for which the normalization is obtained from data in the $M_3$ analysis (see Sect. 8). In the $M_{\ell \mathrm{b}}$ analysis the multijet background is reduced to a negligible fraction (see Sect. 4) and thus not considered further.

Pileup signals, i.e. extra activity due to additional pp interactions in the same bunch crossing, are incorporated by simulating additional interactions with a multiplicity matching the one inferred from data. The CMS detector response is modeled using Geant4 [52]. The simulated events are processed by the same reconstruction software as the collision data.

## Reconstruction and event selection

This analysis focuses on the selection of $\mathrm{t}\overline{\mathrm{t}}$ lepton+jets decays in the muon and electron channels, with similar selection requirements applied for the two channels. Muons, electrons, photons, and neutral and charged hadrons are reconstructed and identified by the CMS particle-flow (PF) algorithm [53, 54]. The energy of muons is obtained from the corresponding track momentum using the combined information of the silicon tracker and the muon system [55]. The energy of electrons is determined from a combination of the track momentum in the tracker, the corresponding cluster energy in the electromagnetic calorimeter, and the energy sum of all bremsstrahlung photons associated to the track [56]. The vertex with the largest $p_{\mathrm {T}} ^2$ sum of the tracks associated to it is chosen as primary vertex.

Candidate $\mathrm{t}\overline{\mathrm{t}}$ events are first accepted by dedicated triggers requiring at least one muon or electron. Lepton isolation requirements are applied to improve the purity of the selected sample. At the trigger level the relative muon isolation, the sum of transverse momenta of other particles in a cone of size $\Delta R =\sqrt{\smash [b]{(\Delta \phi )^2+(\Delta \eta )^2} } = 0.4$ around the direction of the candidate muon divided by the muon transverse momentum, is required to be less than 0.2. Similarly, for electrons, the corresponding requirement is less than 0.3 in a cone of size 0.3. Events with a muon in the final state are triggered on the presence of a muon candidate with $p_{\mathrm {T}} > 24\,\text {GeV}$ and $|\eta | <2.1$. Events with an electron candidate with $|\eta | < 2.5$ are accepted by triggers requiring an electron with $p_{\mathrm {T}} > 27\,\text {GeV}$.

Tighter $p_{\mathrm {T}}$ requirements are applied in the offline selections. Muons are required to have a good quality [55] track with $p_{\mathrm {T}} > 25 \,\text {GeV}$ and $|\eta | <2.1$. Electrons are identified using a combination of the shower shape information and track-electromagnetic cluster matching [56], and are required to have $p_{\mathrm {T}}\ > 32\,\text {GeV}$ and $|\eta | < 2.5$, with the exclusion of the transition region between the barrel and endcap electromagnetic calorimeter, $1.44< |\eta | < 1.57$. Electrons identified to come from photon conversions [56] are vetoed. Correction factors for trigger and lepton identification efficiencies have been determined with a tag-and-probe method [57] from data/simulation comparison as a function of the lepton $p_{\mathrm {T}}$ and $\eta$, and are applied to the simulation.

Signal events are required to have at least one pp interaction vertex, successfully reconstructed from at least four tracks, within limits on the longitudinal and radial coordinates [58], and exactly one muon, or electron, with an origin consistent with the reconstructed vertex within limits on the impact parameters. Since the lepton from the $\mathrm {W}$ boson decay is expected to be isolated from other activity in the event, isolation requirements are applied. A relative isolation is defined as $I_{\text {rel}} =(I_{\text {charged}} + I_{\text {photon}} + I_{\text {neutral}}) / p_{\mathrm {T}}$, where $p_{\mathrm {T}}$ is the transverse momentum of the lepton and $I_{\text {charged}}$, $I_{\text {photon}}$, and $I_{\text {neutral}}$ are the sums of the transverse energies of the charged particles, the photons, and the neutral particles not identified as photons, in a cone $\Delta R < 0.4\,(0.3)$ for muons (electrons) around the lepton direction, excluding the lepton itself. The relative isolation $I_{\text {rel}}$ is required to be less than 0.12 for muons and 0.10 for electrons. Events with more than one lepton candidate with relaxed requirements are vetoed in order to reject $\mathrm{Z}$ boson or $\mathrm{t}\overline{\mathrm{t}}$ decays into dileptons.

**Fig. 1:**
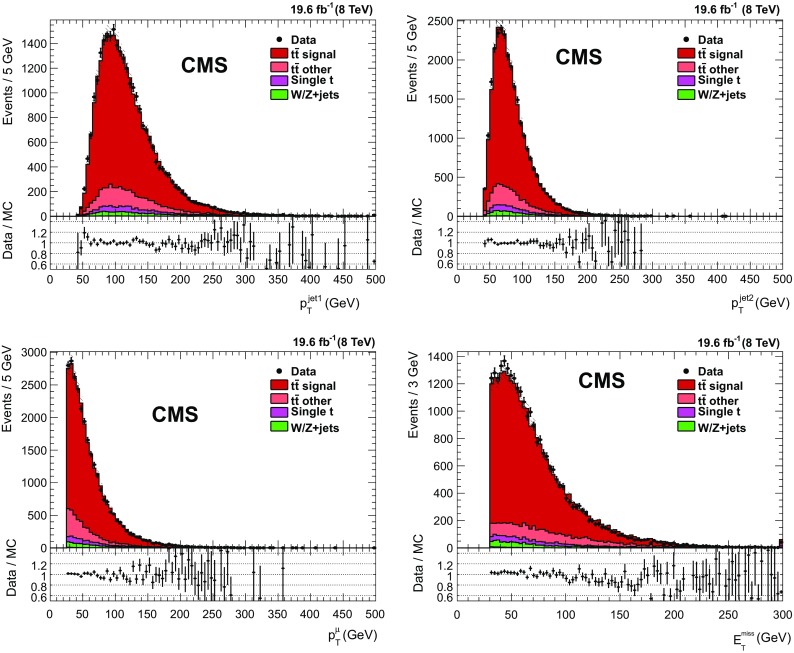
Transverse momentum distributions of the first- and second-leading jet (*top*), the muon and $E_{\mathrm {T}}^{\text {miss}}$ distribution (*bottom*) for all relevant processes in the muon+jets channel with the requirement of at least one $\mathrm{b}$-tagged jet. The simulation is normalized to the standard model cross section values and $p_{\mathrm {T}}$-reweighting is applied to the $\mathrm{t}\overline{\mathrm{t}}$ contribution. The multijet background is negligible and not shown. The distributions are already corrected for the $\mathrm{b}$ tagging efficiency scale factor. The hashed area shows the uncertainty in the luminosity measurement and the $\mathrm{b}$ tagging systematic uncertainty. The last bin includes the overflow. The ratio between data and simulation is shown in the lower panels for bins with non-zero entries.eps

The missing energy in the transverse plane ($E_{\mathrm {T}}^{\text {miss}}$) is defined as the magnitude of the projection on the plane perpendicular to the beams of the vector sum of the momenta of all PF candidates. It is required to be larger than 30$\,\text {GeV}$ in the muon channel and larger than 40$\,\text {GeV}$ in the electron channel, because of the larger multijet background.

Jets are clustered from the charged and neutral particles reconstructed with the PF algorithm, using the anti-$k_{\mathrm {T}}$ jet algorithm [59] with a radius parameter of 0.5. Particles identified as isolated muons or electrons are not used in the jet clustering. Jet energies are corrected for nonlinearities due to different responses in the calorimeters and for the differences between measured and simulated responses [60]. Furthermore, to account for extra activity within a jet cone due to pileup, jet energies are corrected [53, 54] for charged hadrons that belong to a vertex other than the primary vertex, and for the amount of pileup expected in the jet area from neutral jet constituents.

**Fig. 2:**
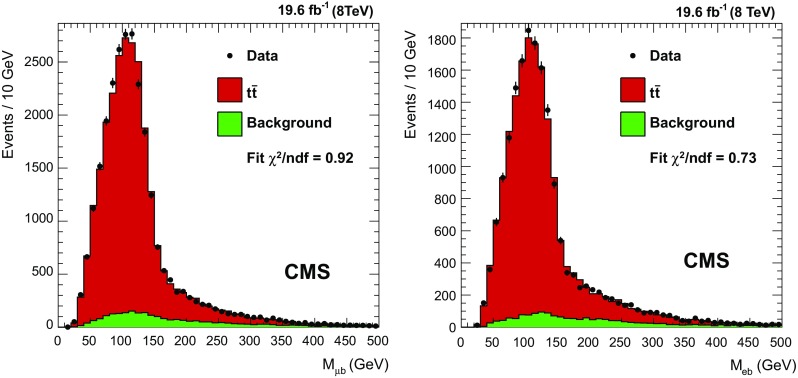
Distributions of the lepton-jet mass in the muon+jets (*left*) and electron+jets (*right*) channels, rescaled to the fit results

At least four jets are required with $p_{\mathrm {T}} > 40\,\text {GeV}$ and $|\eta | < 2.5$. An additional global calibration factor of the jet energy scale is obtained by fitting the $\mathrm {W}$ boson mass distribution in the data and in the simulation. The scale factor is determined as the ratio of the $\mathrm {W}$ boson mass reconstructed from non-$\mathrm{b}$-tagged jet pairs in data and in the simulation. This scale correction is applied in the simulation to all jets before the selection requirements are implemented. It largely reduces the systematic uncertainty related to the jet energy scale, discussed in Sect. 6.

To reduce contamination from background processes, at least one of the jets has to be identified as a $\mathrm{b}$ jet. The $\mathrm{b}$ tagging algorithm used is the “combined secondary vertex” (CSV) algorithm at the medium working point [26, 27], corresponding to a misidentification probability of about 1% for light-parton jets (mistag rate) and an efficiency for $\mathrm{b}$ jets in the range 60–70% depending on the jet $p_{\mathrm {T}}$ and pseudorapidity. Figure 1 shows kinematic distributions after applying the $\mathrm{b}$ tagging requirement. Good agreement between data and simulation is observed.

The $M_{\ell \mathrm{b}}$ analysis uses control samples in data for the estimation of the b tagging efficiency, as described in Refs. [26–28]. Among the four leading jets, three are assigned to the hadronically decaying top quark through a $\chi ^2$ sorting algorithm using top quark and $\mathrm {W}$ boson mass constraints. The remaining fourth jet is the $\mathrm{b}$ jet candidate assigned to the leptonically decaying top quark. The $\mathrm{b}$ tagging algorithm is only applied to this $\mathrm{b}$ jet candidate.

Owing to differences in the triggers and in the centre-of-mass energies, in the 7$\,\text {TeV}$ analysis slightly different selection criteria are applied on the lepton $p_{\mathrm {T}}$ and $E_{\mathrm {T}}^{\text {miss}}$. The muon transverse momentum is required to be larger than 26$\,\text {GeV}$, while the electron $p_{\mathrm {T}}$ has to be larger than 30$\,\text {GeV}$. No explicit $E_{\mathrm {T}}^{\text {miss}}$ requirement is needed in the muon channel. Events with $E_{\mathrm {T}}^{\text {miss}} > 30\,\text {GeV}$ are selected in the electron channel.

## Visible and total cross section measurements

The number of $\mathrm{t}\overline{\mathrm{t}}$ events is determined with a binned maximum-likelihood fit of distributions (templates), describing signal and background processes, to the data sample passing the final selection, by fitting $M_{\ell \mathrm{b}}$, the invariant mass distribution of the $\mathrm{b}$ jet and the lepton.

The $\mathrm{t}\overline{\mathrm{t}}$ visible ($\sigma _{\mathrm{t}\overline{\mathrm{t}}}^{\text {vis}}$) and total ($\sigma _{\mathrm{t}\overline{\mathrm{t}}}$) production cross sections are extracted from the number of $\mathrm{t}\overline{\mathrm{t}}$ events observed in the data using the equations

1 $$\begin{aligned} \sigma _{\mathrm{t}\overline{\mathrm{t}}}^{\text {vis}} = \frac{N_{\mathrm{t}\overline{\mathrm{t}}}}{ L \, \varepsilon _{\mathrm{t}\overline{\mathrm{t}}}},\quad \sigma _{\mathrm{t}\overline{\mathrm{t}}} = \frac{\sigma _{\mathrm{t}\overline{\mathrm{t}}}^{\text {vis}}}{A} , \end{aligned}$$

where $N_{\mathrm{t}\overline{\mathrm{t}}}$ is the number of $\mathrm{t}\overline{\mathrm{t}}$ events (including both signal events from the lepton+jets channel considered and events from other decay channels) extracted from the fit, *L* is the integrated luminosity, *A* is the $\mathrm{t}\overline{\mathrm{t}}$ acceptance, and $\varepsilon _{\mathrm{t}\overline{\mathrm{t}}}$ is the $\mathrm{t}\overline{\mathrm{t}}$ selection efficiency within the acceptance requirements outlined in the next section. Results are presented for both the visible and total cross section, in order to separate experimental uncertainties from theoretical assumptions as much as possible.

One template is used for $\mathrm{t}\overline{\mathrm{t}}$ events (both for the $\mathrm{t}\overline{\mathrm{t}}$ signal events and the other $\mathrm{t}\overline{\mathrm{t}}$ events passing the selection criteria) and one template for all background processes ($\mathrm {W}/\mathrm{Z}$+jets and single top quark production). The fit is performed in the range 0–500$\,\text {GeV}$. Figure 2 shows the results for the fit to the data distributions in the muon and electron channels.

### Acceptance

The $\mathrm{t}\overline{\mathrm{t}}$ acceptance *A* corresponding to the visible phase space depends on the theoretical model and it is determined at the generator level by requiring the presence of exactly one lepton, one neutrino, and at least four jets, passing $p_{\mathrm {T}}$ and $|\eta |$ selection criteria similar to the ones delineated in Sect. 4. For simplicity a single acceptance definition, corresponding to the tightest selection criteria, is used for both channels at each centre-of-mass energy: exactly one muon, or electron, with $p_{\mathrm {T}} > 32\,\text {GeV}$ and $|\eta | < 2.1$, one neutrino with $p_{\mathrm {T}} > 40\,\text {GeV}$, and at least four jets with $p_{\mathrm {T}} > 40\,\text {GeV}$ and $|\eta | < 2.5$.

The acceptance values include contributions from other $\mathrm{t}\overline{\mathrm{t}}$ decay channels, in particular from the dilepton channel, at the level of about 9%.

**Table 1:** Average acceptance values for the muon and electron channels obtained with MadGraph and powheg at $\sqrt{s} = 7$ and 8$\,\text {TeV}$, without and with top quark $p_{\mathrm {T}}$-reweighting applied. The statistical uncertainty is 0.0004, i.e. below 3%. The theoretical uncertainties are at the level of 2%, as discussed in the text

	*A* ($\sqrt{s} = 7\,\text {TeV}$)		*A* ($\sqrt{s} = 8\,\text {TeV}$)	
	No rew.	With rew.	No rew.	With rew.
MadGraph	0.0158	0.0156	0.0166	0.0162
powheg	0.0151	0.0149	0.0163	0.0161

**Table 2:** Signal selection efficiencies, at $\sqrt{s} = 8\,\text {TeV}$, determined from simulation using MadGraph.The non-reweighted acceptance from Table 1 is used. The relative statistical uncertainty on $\varepsilon _{\mathrm{t}\overline{\mathrm{t}}}$ is below 3%

Channel	$\varepsilon _{\mathrm{t}\overline{\mathrm{t}}}$ ($\sqrt{s} = 7\,\text {TeV}$) (%)	$A \varepsilon _{\mathrm{t}\overline{\mathrm{t}}}$ ($\sqrt{s} = 7\,\text {TeV}$) (%)	$\varepsilon _{\mathrm{t}\overline{\mathrm{t}}}$ ($\sqrt{s} = 8\,\text {TeV}$) (%)	$A \varepsilon _{\mathrm{t}\overline{\mathrm{t}}}$ ($\sqrt{s} = 8\,\text {TeV}$) (%)
$\mu$+jets	37	0.58	32	0.53
$\mathrm {e}$+jets	22	0.36	21	0.35

The acceptance values are provided in Table 1 for the two generators used in this analysis, MadGraph and powheg. The acceptance values are in agreement at the 1–2% level at 8$\,\text {TeV}$ and at better than 5% at 7$\,\text {TeV}$. This different level of agreement is due to the fact that the common acceptance definition described above corresponds the tightest $p_{\mathrm {T}}$ criteria, i.e. to the $p_{\mathrm {T}}$ requirements of the electron channel at $\sqrt{s} = 8$ $\,\text {TeV}$. The reweighted acceptance is determined as the number of reweighted $\mathrm{t}\overline{\mathrm{t}}$ events in the visible phase space, i.e. the sum of the weights, divided by the total number of (non-reweighted) $\mathrm{t}\overline{\mathrm{t}}$ events.

The statistical uncertainty in the acceptance calculations is below 3%. The theoretical systematic uncertainties evaluated by varying the PDFs (Sect. 6) or the matching thresholds are in the range 0.1–0.2%. Variation of the factorization and renormalization scale induces a variation of up to 2% in the acceptance. These variations are already included in the systematic uncertainties quoted in Sect. 6.

In the following, top quark $p_{\mathrm {T}}$-reweighting [45, 46] is always applied to the visible phase space as it provides a better agreement between data and simulation. On the other hand, given that the event weights were only determined in the phase space corresponding to the experimental selection, they have not been used for the extrapolation to the total cross section. Therefore, the non-reweighted acceptance is used to determine the total cross section. However, rescaling by the ratio of the values provided in Table 1 would allow a determination of the total cross section with the reweighted acceptance. The visible cross section does not depend on the acceptance *A*.

### Selection efficiency

The selection efficiency within the acceptance, $\varepsilon _{\mathrm{t}\overline{\mathrm{t}}}$, is reported in Table 2. It is determined from the $p_{\mathrm {T}}$-reweighted MadGraph simulated sample as the number of events passing the selection criteria outlined in Sect. 4, over the number of events passing the acceptance requirements defined above. The selection efficiency includes the effects of trigger requirements, lepton and jet identification criteria, and $\mathrm{b}$ tagging efficiency, which is directly determined from data. A signal selection efficiency within acceptance of $32\%$ in the muon channel and $21\%$ in the electron channel is determined. Similar values (37 and 22%, respectively) are obtained at $\sqrt{s} = 7\,\text {TeV}$. For the muon channel the common acceptance requirements used for both channels are tighter than the selection requirements, thus the muon channel efficiency is significantly larger than the electron channel efficiency. The $\mathrm{t}\overline{\mathrm{t}}$ selection efficiency, $A \varepsilon _{\mathrm{t}\overline{\mathrm{t}}}$, is the number of selected $\mathrm{t}\overline{\mathrm{t}}$ events out of all produced $\mathrm{t}\overline{\mathrm{t}}$ pairs, in all decay channels.

## Systematic uncertainties

Systematic uncertainties are determined by varying each source within its estimated uncertainty and by propagating the variation to the cross section measurements. Template shapes and signal efficiencies are varied together according to the systematic uncertainty considered. The uncertainty is given by the shift in the fitted cross section and is cross-checked by repeating its estimation with pseudo-experiments using simulation. The systematically varied template shapes are fit to pseudo-data generated using the nominal template shapes and normalizations. The validation with pseudo-experiments shows that the fit performs as expected. All systematic uncertainties, except the ones related to $\mathrm{b}$ tagging and to the estimation of the multijet background, are common to both the $M_{\ell \mathrm{b}}$ and the $M_3$ measurements.

The effect of uncertainties in the JES is evaluated by varying the JES within the $p_{\mathrm {T}}$- and $\eta$-dependent uncertainties given in Ref. [60]. The final JES of the simulation is matched to that in data by applying an additional global correction factor $\alpha$ to all jet momenta before selection. The $\alpha$ calibration values are individually determined for nominal conditions and for each of the variations related to JES and JER. In addition to the selection described in Sect. 4, two $\mathrm{b}$-tagged jets are required in order to increase the signal purity. The mass of the hadronically decaying $\mathrm {W}$ boson is reconstructed as the dijet invariant mass from all combinations of non $\mathrm{b}$-tagged jets. The dijet invariant mass distributions are fitted in data and in simulation with a function describing the $\mathrm {W}$ boson signal peak and the dijet combinatorial background. The $\alpha$ values are determined as the ratios of the fitted $\mathrm {W}$ boson masses in data and in simulation. In the $M_{\ell \mathrm{b}}$ analysis $\alpha = 1.011 \pm 0.004$ is obtained with the nominal samples both in the muon and electron channels, with variations of the order of ±1.5% for the samples with down and up variations of the JES. The same values are determined by the $M_3$ analysis. This additional calibration reduces the size of the JES systematic uncertainty by approximately 60%. The JES uncertainty, reported in Table 3, consists of several sources, all propagated individually. Details of the individual contributions are explained in [61].

**Table 3:** Components (in %) of the JES uncertainty at 8$\,\text {TeV}$ in the muon and electron channels. The correlation coefficients used in their combination are also shown

Source	$\mu$+jets	$\mathrm {e}$+jets	Correlation
Absolute scale	${\pm }0.33$	${\pm }0.40$	0.0
Global jet scale factor $\alpha$	${\pm }0.59$	${\pm }0.39$	0.0
Relative FSR	${\pm }0.46$	${\pm }0.41$	1.0
Relative $p_{\mathrm {T}}$	${\pm }0.67$	${\pm }0.57$	1.0
Flavour JES	${\pm }1.84$	${\pm }1.79$	1.0
Flavour JES fragmentation	${\pm }0.50$	${\pm }0.46$	1.0
Flavour JES semileptonic BR	${\pm }0.11$	${\pm }0.16$	1.0
High-$p_{\mathrm {T}}$ extra	${\pm }0.18$	${\pm }0.23$	1.0
Single pion	${\pm }0.21$	${\pm }0.27$	1.0
Pileup	${\pm }0.35$	${\pm }0.31$	1.0
Time	${\pm }0.17$	${\pm }0.24$	1.0
Total JES	${\pm }2.23$	${\pm }2.13$	0.9

The impact of the jet energy resolution (JER) is estimated by applying $\eta$-dependent variations with an average of $\pm 10\%$. The JES and JER variations are propagated to the $E_{\mathrm {T}}^{\text {miss}}$. In addition, the contribution to $E_{\mathrm {T}}^{\text {miss}}$  arising from energy depositions not contained in jets is varied by $\pm 10\%$ [60]. The uncertainty related to the pileup modelling is determined by propagating a $\pm 5\%$ variation [62] to the central value of the inelastic cross section. Variations in the composition of the main background processes, $\mathrm {W}$+jets and $\mathrm{Z}$+jets, are conservatively evaluated by varying independently their cross sections by $\pm 30\%$ [63–65]. Additional uncertainties on the heavy flavour component in $\mathrm {W}/\mathrm{Z}$+jets production are not explicitly taken into account and are assumed to be covered by the 30% uncertainty. The variation of the normalization of the single top quark background by 30% gives a negligible contribution. The trigger efficiency and lepton identification correction factors are determined with a tag-and-probe method [57] in dilepton events and are varied within their $p_{\mathrm {T}}$- and $\eta$-dependent uncertainties.

Uncertainties from the $\mathrm{b}$ tagging efficiency and mistag rate are evaluated in the $M_3$ analysis by varying the correction factors within their uncertainties [26, 27] quoted in Sect. 8. In the $M_{\ell \mathrm{b}}$ analysis, on the other hand, the $\mathrm{b}$ tagging efficiency for $\mathrm{b}$ jets is measured from data, using the technique described in Refs. [26–28], on the same selected event sample as that for the cross section determination, but before $\mathrm{b}$ tagging. The $M_{\ell \mathrm{b}}$ variable is used not only as a cross section estimator, but also as a $\mathrm{b}$ tagging discriminator. The statistical and systematic uncertainties in the $\mathrm{b}$ tagging and mistag efficiencies are propagated to the statistical and systematic uncertainties in the cross section measurements. For this reason the statistical uncertainty obtained by the $M_{\ell \mathrm{b}}$ analysis is larger than the one of the $M_3$ analysis. A systematic uncertainty is assigned to the choice, based on simulation, of the $\mathrm{b}$-enriched (for $M_{\ell \mathrm{b}}$ values below 140$\,\text {GeV}$) and of the $\mathrm{b}$-depleted (for $M_{\ell \mathrm{b}}$ in the range 140–240$\,\text {GeV}$) regions, by shifting the windows by $\pm 30\,\text {GeV}$. Since the $\mathrm{b}$ tagging efficiency and mistag rate are derived from data and since they are re-determined when evaluating the effect of the various systematic uncertainties, no additional uncertainties are included. The method is shown [26–28] to be stable for different $\mathrm{b}$ tagging algorithms and working points.

**Table 4:** Overview of the systematic uncertainties in the measurement of the $\mathrm{t}\overline{\mathrm{t}}$ cross sections at 8$\,\text {TeV}$, both for the total and the visible cross sections. For the “signal modelling” uncertainty the larger between the matrix element (ME) and parton shower (PS) uncertainties is taken, as explained in Sect. 6. The correlations assumed for the combination of the muon and electron channels are also given

Systematic uncertainty	8$\,\text {TeV}$			
	$\mu$+jets (%)	$\mathrm {e}$+jets (%)	corr.	comb.(%)
Jet energy scale	${\pm }2.2$	${\pm }2.1$	0.9	${\pm }2.2$
Jet energy resolution	${\pm }0.8$	${\pm }0.9$	1.0	${\pm }0.8$
$E_{\mathrm {T}}^{\text {miss}}$ unclustered energy	${\pm }0.1$	${\pm }0.3$	1.0	${\pm }0.1$
Pileup	${\pm }0.5$	${\pm }0.4$	1.0	${\pm }0.5$
Lepton ID / Trigger eff. corrections	${\pm }0.4$	${\pm }0.5$	0.0	${\pm }0.5$
$\mathrm{b}$ tagging method	${\pm }0.3$	${\pm }0.7$	1.0	${\pm }0.3$
Background composition	${\pm }0.2$	${\pm }0.3$	1.0	${\pm }0.2$
Factorization/renormalization scales	${\pm }1.7$	${\pm }2.6$	1.0	${\pm }1.7$
ME-PS matching threshold	${\pm }1.3$	${\pm }2.3$	1.0	${\pm }1.2$
Top quark $p_{\mathrm {T}}$-reweighting	${\pm }1.1$	${\pm }1.2$	1.0	${\pm }1.1$
Signal modelling for $\sigma _{\mathrm{t}\overline{\mathrm{t}}}\,\,(\sigma _{\mathrm{t}\overline{\mathrm{t}}}^{\text {vis}})$	${\pm }4.4\,({\pm }2.2)$	${\pm }4.4\,({\pm }2.4)$	1.0	${\pm }4.4\,({\pm }2.3)$
PDF uncertainties	${\pm }2.1$	${\pm }1.9$	1.0	${\pm }2.1$
Sum for $\sigma _{\mathrm{t}\overline{\mathrm{t}}}\,\,(\sigma _{\mathrm{t}\overline{\mathrm{t}}}^{\text {vis}})$	${\pm }6.0 \,({\pm }4.6)$	${\pm }6.5\,({\pm }5.4)$		${\pm }6.0\,\,({\pm }4.7)$
Integrated luminosity	${\pm }2.6$	${\pm }2.6$	1.0	${\pm }2.6$
Total for $\sigma _{\mathrm{t}\overline{\mathrm{t}}}\,\,(\sigma _{\mathrm{t}\overline{\mathrm{t}}}^{\text {vis}})$	${\pm }6.5\, ({\pm }5.3)$	${\pm }7.0\,({\pm }6.0)$		${\pm }6.5\,\,({\pm }5.3)$

**Table 5:** Overview of the systematic uncertainties in the measurement of the $\mathrm{t}\overline{\mathrm{t}}$ cross sections at 7$\,\text {TeV}$, both for the total and the visible cross sections. For the “signal modelling” uncertainty the larger between the matrix element (ME) and parton shower (PS) uncertainties is taken, as explained in Sect. 6. The correlations assumed for the combination of the muon and electron channels are also shown.

Systematic uncertainty	7$\,\text {TeV}$			
	$\mu$+jets (%)	e+jets (%)	corr.	comb. (%)
Jet energy scale	${\pm }4.8$	${\pm }5.2$	0.9	${\pm }4.4$
Jet energy resolution	${\pm }1.4$	${\pm }1.1$	1.0	${\pm }1.1$
$E_{\mathrm {T}}^{\text {miss}}$ unclustered energy	<0.05	${\pm }0.3$	1.0	${\pm }0.2$
Pileup	${\pm }0.4$	${\pm }0.6$	1.0	${\pm }0.5$
Lepton ID/trigger eff. corrections	${\pm }1.4$	${\pm }1.7$	0.0	${\pm }0.8$
$\mathrm{b}$ tagging method	${\pm }0.5$	${\pm }0.6$	1.0	${\pm }0.6$
Background composition	${\pm }0.5$	${\pm }0.4$	1.0	${\pm }0.5$
Factorization/renormalization scales	${\pm }3.7$	${\pm }0.4$	1.0	${\pm }2.1$
ME-PS matching threshold	${\pm }2.0$	${\pm }1.7$	1.0	${\pm }1.8$
Top quark $p_{\mathrm {T}}$-reweighting	${\pm }1.1$	${\pm }1.2$	1.0	${\pm }1.1$
Signal modelling for $\sigma _{\mathrm{t}\overline{\mathrm{t}}}\,\,(\sigma _{\mathrm{t}\overline{\mathrm{t}}}^{\text {vis}})$	${\pm }4.4\,({\pm }2.2)$	${\pm }4.4\,({\pm }2.4)$	1.0	${\pm }4.4\,({\pm }2.3)$
PDF uncertainties	${\pm }2.3$	${\pm }1.9$	1.0	${\pm }2.2$
Sum for $\sigma _{\mathrm{t}\overline{\mathrm{t}}}\,\,(\sigma _{\mathrm{t}\overline{\mathrm{t}}}^{\text {vis}})$	${\pm }8.4\,({\pm }7.5)$	${\pm }7.7\,({\pm }6.8)$		${\pm }7.4\,({\pm }6.4)$
Integrated luminosity	${\pm }2.2$	${\pm }2.2$	1.0	${\pm }2.2$
Total for $\sigma _{\mathrm{t}\overline{\mathrm{t}}}\,\,(\sigma _{\mathrm{t}\overline{\mathrm{t}}}^{\text {vis}})$	${\pm }8.7\,({\pm }7.8)$	${\pm }8.0\,({\pm }7.1)$		${\pm }7.7\,({\pm }6.7)$

Theoretical uncertainties are taken from detailed studies performed on simulated samples. They include the common factorization and renormalization scales, which are varied by a factor of 1/4 and 4 from the default value equal to the $Q^2$ for the $\mathrm{t}\overline{\mathrm{t}}$ or $\mathrm {W}/\mathrm{Z}$+jet events. The effect of the jet-parton matching threshold on $\mathrm{t}\overline{\mathrm{t}}$ and $\mathrm {W}$+jets events is studied by varying the threshold used for matching the matrix element level to the particles created in the parton showering by a factor of 0.5 or 2. Uncertainties from the choice of PDF are evaluated by using the Hessian method [66] with the parameters of the CTEQ6.6 PDF set [67]. Other PDF sets (including their uncertainties) yield very similar results. The PDFs and their uncertainties are determined from a fit to collision data yielding the Hessian matrix. Each of the 22 eigenvectors obtained by diagonalizing the matrix is varied within its uncertainties. The differences with respect to the nominal prediction are determined independently for each eigenvector and are added in quadrature. The systematic uncertainty due to the top quark $p_{\mathrm {T}}$-reweighting procedure described in Sect. 3 is evaluated as the difference with respect to the measurement obtained with the non-reweighted sample. Only the variation due to the template shape is considered, as the correction is meant to modify the shape only.

A “signal modelling” uncertainty is attributed to the choice of the generators. It comprises changes in matrix element and parton shower implementation. The effect of the matrix element generator is evaluated by using powheg (instead of MadGraph) interfaced to pythia, while the parton shower modelling is evaluated with powheg and herwig instead of powheg and pythia. Regarding the two corresponding uncertainties, the former is always positive and the latter is always negative. For 7$\,\text {TeV}$ the same values determined for 8$\,\text {TeV}$ are used. As discussed in Sect. 7, the “signal modelling” uncertainty is symmetrized by taking the larger of the two contributions ($\pm 4.4\%$).

An uncertainty of 2.6% [30] (2.2% [31]) is assigned to the determination of the 2012 (2011) integrated luminosity. The resulting effects from all sources are added in quadrature. Tables 4 and 5 provide an overview of the contributions to the systematic uncertainty on the combined cross section measurements in the $M_{\ell \mathrm{b}}$ measurements at 7 and 8$\,\text {TeV}$.

**Table 6:** Visible cross section measurements at $\sqrt{s} =$ 7 and 8$\,\text {TeV}$ with the reference analysis $M_{\ell \mathrm{b}}$ and the alternative analysis $M_3$ (described in Sect. 8). Results obtained for $m_{\mathrm{t}}=172.5\,\text {GeV}$ with MadGraph and with powheg are shown. The uncertainties are in the order: statistical, systematic, and due to the luminosity determination

Analysis	Generator	Channel	$\sigma _{\mathrm{t}\overline{\mathrm{t}}}^{\text {vis}}$ at $\sqrt{s} = 8\,\text {TeV}$
$M_{\ell \mathrm{b}}$	MadGraph	$\mu$+jets	$3.80 \pm 0.06 \pm 0.18 \pm 0.10\text { pb}$
		$\mathrm {e}$+jets	$3.90 \pm 0.07 \pm 0.21 \pm 0.10\text { pb}$
		Combined	$3.80 \pm 0.06 \pm 0.18 \pm 0.10\text { pb}$
$M_{\ell \mathrm{b}}$	powheg	Combined	$3.83 \pm 0.06 \pm 0.18 \pm 0.10\text { pb}$
$M_3$	MadGraph	$\mu$+jets	$3.79 \pm 0.05 \pm 0.24 \pm 0.10\text { pb}$
		$\mathrm {e}$+jets	$3.75 \pm 0.04 \pm 0.26 \pm 0.10\text { pb}$
		Combined	$3.78 \pm 0.04 \pm 0.25 \pm 0.10\text { pb}$
$M_3$	powheg	Combined	$3.88 \pm 0.05 \pm 0.27 \pm 0.10\text { pb}$
Analysis	Generator	Channel	$\sigma _{\mathrm{t}\overline{\mathrm{t}}}^{\text {vis}}$ at $\sqrt{s} = 7\,\text {TeV}$
$M_{\ell \mathrm{b}}$	MadGraph	$\mu$+jets	$2.48 \pm 0.09 \pm 0.19 \pm 0.06\text { pb}$
		$\mathrm {e}$+jets	$2.62 \pm 0.10 \pm 0.18 \pm 0.06\text { pb}$
		Combined	$2.55 \pm 0.09 \pm 0.18 \pm 0.06\text { pb}$

**Table 7:** Total cross section measurements at $\sqrt{s} =$ 7 and 8$\,\text {TeV}$ with the reference analysis $M_{\ell \mathrm{b}}$ and the alternative analysis $M_3$ (described in Sect. 8). Results obtained for $m_{\mathrm{t}}=172.5\,\text {GeV}$ with MadGraph and with powheg are shown. The uncertainties are in the order: statistical, systematic, and due to the luminosity determination.

Analysis	Generator	Channel	$\sigma _{\mathrm{t}\overline{\mathrm{t}}}$ at $\sqrt{s} = 8\,\text {TeV}$
$M_{\ell \mathrm{b}}$	MadGraph	$\mu$+jets	$228.9 \pm 3.4 \pm 13.7 \pm 6.0\text { pb}$
		$\mathrm {e}$+jets	$234.6 \pm 3.9 \pm 15.2 \pm 6.2\text { pb}$
		Combined	$228.5 \pm 3.8 \pm 13.7 \pm 6.0\text { pb}$
$M_{\ell \mathrm{b}}$	powheg	Combined	$237.1 \pm 3.9 \pm 14.2 \pm 6.2\text { pb}$
$M_3$	MadGraph	$\mu$+jets	$228.7 \pm 2.6 \pm 19.0 \pm 6.0\text { pb}$
		$\mathrm {e}$+jets	$225.8 \pm 2.4 \pm 19.1 \pm 5.9\text { pb}$
		Combined	$227.1 \pm 2.5 \pm 19.1 \pm 6.0\text { pb}$
$M_3$	powheg	Combined	$238.4 \pm 2.8 \pm 20.0 \pm 6.2\text { pb}$
Analysis	Generator	Channel	$\sigma _{\mathrm{t}\overline{\mathrm{t}}}$ at $\sqrt{s} = 7\,\text {TeV}$
$M_{\ell \mathrm{b}}$	MadGraph	$\mu$+jets	$157.7 \pm 5.5 \pm 13.2 \pm 3.4\text { pb}$
		$\mathrm {e}$+jets	$165.8 \pm 6.5 \pm 12.8 \pm 3.6\text { pb}$
		Combined	$161.7 \pm 6.0 \pm 12.0 \pm 3.6\text { pb}$

## Results and combination

The results in the muon and electron channels, shown in Tables 6 and 7, are in good agreement. The combination of the channel results is performed using the best linear unbiased estimator (BLUE) method [68–70]. Asymmetric systematic uncertainties are symmetrized for the use with BLUE by taking half of the full range, except for the “signal modelling” uncertainty, where the maximum, 4.4%, is taken for $\sigma _{{\mathrm{t}\overline{\mathrm{t}}}}$. Full correlation is assumed for all systematic uncertainties between the two channels, except for lepton identification and trigger uncertainties, which are assumed to be uncorrelated.

Owing to the additional jet energy calibration from data, a correlation coefficient of 0.9 is obtained for the overall JES uncertainty. This correlation is determined from the correlation coefficients in Table 3 and it is compatible with the value inferred by comparing the combined result with and without the additional calibration. Varying the JES correlation coefficient between 0 and 1 has only a minor effect on the combined results. For example, the total cross section at 8$\,\text {TeV}$ varies by less than 0.5%, and the cross section ratio varies only by approximately 0.1%. A combination based on the relative statistical precision of the two channels would also yield compatible results. Variations of the correlations of other experimental systematic uncertainties have negligible effect on the combined results.

The integrated luminosity and the pileup uncertainties are assumed to be fully correlated between channels at the same centre-of-mass energy, and uncorrelated between 7 and 8$\,\text {TeV}$ for the cross section ratio.

### Results at $\sqrt{s}=8\,\text {TeV}$

The visible cross section obtained from the fit to the $M_{\ell \mathrm{b}}$ distribution, using MadGraph signal templates for $m_{\mathrm{t}} = 172.5\,\text {GeV}$, is

$$\begin{aligned}&\sigma _{\mathrm{t}\overline{\mathrm{t}}}^{\text {vis}} (\text {combined}) \\&\quad = 3.80 \pm 0.06\,\text {(stat)} \pm 0.18\,\text {(syst)} \pm 0.10\,\text {(lumi)} \text { pb}. \end{aligned}$$

The statistical uncertainty includes the contribution from the simultaneous determination of the $\mathrm{b}$ tagging efficiency (see Sect. 6). There is excellent agreement with the measurement of the visible cross section using powheg for the efficiency within the kinematic acceptance selected by this analysis.

Using the acceptance values of Table 1, the visible cross section measurements in the electron and muon channels are first extrapolated to the full phase space and then combined to obtain the following total cross section measurement

$$\begin{aligned}&\sigma _{\mathrm{t}\overline{\mathrm{t}}} (\text {combined}) \\&\quad = 228.5 \pm 3.8\,\text {(stat)} \pm 13.7\,\text {(syst)} \pm 6.0\,\text {(lumi)} \text { pb}. \end{aligned}$$

The measurements are in good agreement with the theoretical prediction

$$\begin{aligned} \sigma ^{\text {th.}}_{\mathrm{t}\overline{\mathrm{t}}}~(8\,\text {TeV}) = 252.9^{+6.4}_{-8.6} \text {(scale)} \pm 11.7 (\mathrm {PDF}{+}\alpha _S)\text { pb} \end{aligned}$$

(see Sect. 3), for $m_{\mathrm{t}}=172.5\,\text {GeV}$.

The BLUE combination yields the following relative weights of the muon and electron channels, and their correlations, respectively. At 8$\,\text {TeV}$ they are: 1.07 (1.09), $-0.07$ ($-0.09$), with correlation coefficient 0.88 (0.91) for the total (visible) cross section, while at 7$\,\text {TeV}$ they are: 0.50 (0.51), 0.50 (0.49), with correlation coefficient 0.71 (0.65). The negative weights of the electron channel in the combination of the total and visible cross section at 8$\,\text {TeV}$ depend on the choice of the JES correlation coefficient (0.9) used in the combination. Smaller JES correlation coefficients (0.5 for the total cross section and 0.2 for the visible cross section) would yield positive BLUE weights. The negative weights causes the combined central value, 228.5$\text { pb}$, to lie outside the interval of the two individual measurements, as summarized in Tables 6 and 7.

Alternatively, using powheg instead of MadGraph, the combined total cross section at 8$\,\text {TeV}$ shifts by $+8.6$ $\text { pb}$. The difference, at the level of less than 4%, is mainly ascribed to the different acceptance for the two generators.

All results are summarized in Tables 6 and 7 for $m_{\mathrm{t}}=172.5\,\text {GeV}$. For powheg the same relative systematic uncertainties as determined for MadGraph are used.

### Dependence on the top quark mass at $\sqrt{s}=8\,\text {TeV}$

Using simulation, the dependence of the measured total cross section on the top quark mass is determined to be linear in the $m_{\mathrm{t}}$ range from 161.5 to 184.5$\,\text {GeV}$. The top quark mass value used for the central results is 172.5$\,\text {GeV}$. The slope values reported in Table 8 can be used to linearly adjust the results in the two channels to other mass values. For $m_{\mathrm{t}}=173.3\,\text {GeV}$ [71] the adjusted results of the two channels yield a combined cross section value

$$\begin{aligned}&\sigma _{\mathrm{t}\overline{\mathrm{t}}} (\text {combined}, m_{\mathrm{t}}=173.3\,\text {GeV})\\&\quad = 227.4 \pm 3.8\,\text {(stat)} \pm 13.7\,\text {(syst)} \pm 6.0\,\text {(lumi)} \text { pb}. \end{aligned}$$

**Table 8:** Slope values for the muon and electron channels obtained with linear fits to the cross section values at $\sqrt{s} = 8\,\text {TeV}$ as a function of the top quark mass. The MadGraph generator is used. The change in sign is due to the acceptance *A*

Channel	Slope (%/$\text {GeV}$) of $\sigma ^{\text {vis}}_{\mathrm{t}\overline{\mathrm{t}}}$	Slope (%/$\text {GeV}$) of $\sigma _{\mathrm{t}\overline{\mathrm{t}}}$
$\mu$+jets	$+0.50 \pm 0.06$	$-0.66 \pm 0.05$
$\mathrm {e}$+jets	$+0.30 \pm 0.04$	$-0.94 \pm 0.05$

### Results at $\sqrt{s}=7\,\text {TeV}$ and cross section ratio

At $\sqrt{s} = 7\,\text {TeV}$ the measured cross section, with MadGraph, is

$$\begin{aligned}&\sigma _{\mathrm{t}\overline{\mathrm{t}}} (\text {combined}) \\&\quad = 161.7 \pm 6.0\,\text {(stat)} \pm 12.0\,\text {(syst)} \pm 3.6\,\text {(lumi)} \text { pb}. \end{aligned}$$

The measurements are in good agreement with the theoretical expectation

$$\begin{aligned} \sigma ^{\text {th.}}_{\mathrm{t}\overline{\mathrm{t}}}~(7\,\text {TeV}) = 177.3^{+4.6}_{-6.0}\,\text {(scale)} \pm 9.0\,(\mathrm {PDF}{+}\alpha _S)\text { pb} \end{aligned}$$

at 7$\,\text {TeV}$, for a top quark mass of 172.5$\,\text {GeV}$.

From the measurements of the total cross section at the two centre-of-mass energies, a cross section ratio $R^{8/7}$ is determined. In the ratio the experimental uncertainties, which are correlated between the two analyses (at $\sqrt{s}=7$ or 8$\,\text {TeV}$, in each channel) cancel out, leading to an improved precision in comparison to the individual measurements at 7 or 8$\,\text {TeV}$. The ratio is first determined in the individual muon ($1.45 \pm 0.09$) and electron ($1.41 \pm 0.09$) channels and then combined. The measured ratio is

$$\begin{aligned} R^{8/7} = 1.43 \pm 0.04\,\text {(stat)} \pm 0.07\,\text {(syst)} \pm 0.05\,\text {(lumi)}. \end{aligned}$$

In the combination of the ratios in the two channels the theoretical uncertainties, and the jet-related uncertainties are assumed to be 100% correlated, except the JES uncertainty, which is taken as 90% correlated. The other experimental uncertainties are assumed to be uncorrelated. The expected values of the cross section ratio, for instance $R^{8/7}_{\text {th.}} = 1.429 \pm 0.001\,\text {(scale)} \pm 0.004\,\mathrm {(PDF)} \pm 0.001\,\mathrm {(\alpha _s)} \pm 0.001\,(m_{\mathrm{t}})$ [2], for the MSTW08 PDF set and for $m_{\mathrm{t}}=173.3\,\text {GeV}$, are in good agreement with the measurement.

## Alternative approach at $\sqrt{s} = 8\,\text {TeV}$ using $M_3$

In the $M_3$ analysis similar requirements for the selection of $\mathrm{t}\overline{\mathrm{t}}$ lepton+jets decays are used, with slightly different $p_{\mathrm {T}}$-threshold values. Only the differences with respect to the main selection are summarized in the following.

At least four jets are required within $|\eta | < 2.5$ and with $p_{\mathrm {T}} > 50$, 40, 30, and 30$\,\text {GeV}$ in the muon channel, and $p_{\mathrm {T}} > 50$, 45, 35, and 30$\,\text {GeV}$ in the electron channel. Slightly tighter $p_{\mathrm {T}}$ selection criteria are applied in the electron channel because of the larger multijet background. Muons are required to have transverse momentum larger than 26$\,\text {GeV}$. In the muon channel no explicit requirement is applied on the missing energy in the transverse plane, while $E_{\mathrm {T}}^{\text {miss}}$ has to be larger than 20$\,\text {GeV}$ in the electron channel.

**Fig. 3:**
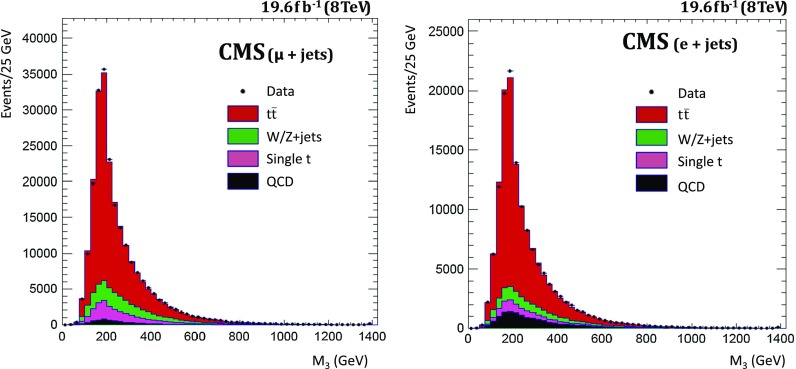
Distributions of the $M_3$ mass in the 8$\,\text {TeV}$ data, for the muon+jets (*left*) and electron+jets (*right*) channels, rescaled to the template likelihood fit results. The last filled bin includes the overflow

The $M_3$ analysis uses a correction factor of $(0.95 \pm 0.02)$ [26, 27] to the simulated events to reproduce the different $\mathrm{b}$ tagging efficiency in data and simulation, and a correction factor of $(1.11 \pm 0.01 \pm 0.12 )$ [26, 27] to take into account the different probability that a light-quark or gluon jet is identified as a $\mathrm{b}$ jet. These correction factors are determined following Refs. [26, 27]. No correction factors are applied in the $M_{\ell \mathrm{b}}$ analysis, where these efficiencies are determined from data.

Different strategies to take into account the multijet background are developed for the $M_{\ell \mathrm{b}}$ and $M_3$ analyses. In the former, this background is reduced to a negligible level thanks to tighter selection requirements on $E_{\mathrm {T}}^{\text {miss}}$ and on the transverse momenta of the third and fourth jets. In the $M_3$ analysis, looser selection cuts are chosen and the multijet background is considered further in the analysis. Since MC simulation can not adequately reproduce the shape and normalization of multijet events, this background is thus estimated from data.

Selected multijet events mostly consist of semileptonic heavy-flavour decays and, in the electron channel, events in which pions in jets are misidentified as electrons. Such events feature lepton candidates not coming from $\mathrm {W}$ boson decays and thus not truly isolated. The shape of the accepted multijet background is extracted from a sideband data sample where leptons have large relative isolation, greater than 0.17 in the muon channel and 0.2 in the electron channel. The data sample is selected such that it is rich in multijet background and poor in $\mathrm{t}\overline{\mathrm{t}}$ signal and in other processes such as $\mathrm {W}$+jets. The remaining $\mathrm{t}\overline{\mathrm{t}}$, $\mathrm {W}$+jets and $\mathrm{Z}$+jets contamination is estimated and subtracted using simulation. Other backgrounds, for example single top quark production, are neglected because of their smaller contributions. The nominal multijet shape is taken as the distribution measured in the sideband after subtracting the components described above.

The template fit is performed with the $M_3$ distribution in the range 0–1400$\,\text {GeV}$. One single template is used for $\mathrm{t}\overline{\mathrm{t}}$ events (both for the $\mathrm{t}\overline{\mathrm{t}}$ signal events and the other $\mathrm{t}\overline{\mathrm{t}}$ events passing the selection requirements) and individual templates are used for each background process. The $\mathrm{t}\overline{\mathrm{t}}$, single top quark, $\mathrm {W}$+jets, and $\mathrm{Z}$+jets templates, used in the likelihood maximization, are taken from simulation, while the multijet template is estimated from data as described above. Because of the similarity between the single top quark and the $\mathrm{t}\overline{\mathrm{t}}$ templates, the single top quark contribution is constrained by a Gaussian distribution of 30% width to its expected value. The choice of the constraint has a negligible effect on the final result. The normalization of the signal and background processes, including the multijet background, is determined by the fit itself. The muon and electron channels are combined with the BLUE method to obtain the quoted combined result. The measured cross section with the $M_3$ template fit is

$$\begin{aligned}&\sigma _{\mathrm{t}\overline{\mathrm{t}}}(\text {combined}) \\&\quad = 227.1 \pm 2.5\,\text {(stat)} \pm 19.1\,\text {(syst)} \pm 6.0\,\text {(lumi)} \text { pb}. \end{aligned}$$

The $M_3$ distributions in the muon and electron channels are shown in Fig. 3. Good agreement is observed between data and the templates. The results are compatible with those of the $M_{\ell \mathrm{b}}$ analysis and are summarized in Tables 6 and 7. The main contributions to the systematic uncertainties of the combined result are, in decreasing order: signal modelling (4.4%), factorization and renormalization scales (2.9%), multijet background subtraction (2.2%), JES (2.1%), PDF (1.6%), and $\mathrm{b}$ tagging efficiency and mistag rate (1.5%). The uncertainty related to the multijet background subtraction is estimated by evaluating two effects. The subtracted $\mathrm{t}\overline{\mathrm{t}}$, $\mathrm {W}$+jets, and $\mathrm{Z}$+jets contaminations are varied by 50%. In addition, we assign an uncertainty to the assumption that the $M_3$ shape does not vary in different regions of relative lepton isolation, by repeating the analysis in six different intervals of the relative lepton isolation.

## Summary

A measurement of the $\mathrm{t}\overline{\mathrm{t}}$ production cross section at $\sqrt{s}=8\,\text {TeV}$ is presented, using the data collected with the CMS detector and corresponding to an integrated luminosity of 19.6$\,\text {fb}^{-\text {1}}$. The analysis is performed in the $\mathrm{t}\overline{\mathrm{t}}$ lepton+jets decay channel with one muon or electron and at least four jets in the final state with at least one $\mathrm{b}$-tagged jet. The $\mathrm{t}\overline{\mathrm{t}}$ cross section is extracted using a binned maximum-likelihood fit of templates from simulated events to the data sample. The results from the two lepton+jets channels are combined using the BLUE method.

Techniques based on control samples in data are used to determine the $\mathrm{b}$ tagging efficiency and to calibrate the jet energy scale. These techniques allow for a better determination of the corresponding systematic uncertainties, particularly for the JES, which is a dominant source of experimental uncertainty.

In the kinematic range defined in the simulation with exactly one muon, or electron, with $p_{\mathrm {T}} > 32\,\text {GeV}$ and $|\eta | < 2.1$, one neutrino with $p_{\mathrm {T}} > 40~$GeV, and at least four jets with $p_{\mathrm {T}} > 40\,\text {GeV}$ and $|\eta | < 2.5$, the measured visible $\mathrm{t}\overline{\mathrm{t}}$ cross section at $\sqrt{s}=8\,\text {TeV}$ is $3.80 \pm 0.06\,\text {(stat)} \pm 0.18\,\text {(syst)} \pm 0.10\,\text {(lumi)} \text { pb}.$

Using the MadGraph generator for the extrapolation to the full phase space, the total $\mathrm{t}\overline{\mathrm{t}}$ cross section at 8$\,\text {TeV}$ is $228.5 \pm 3.8\,\text {(stat)} \pm 13.7\,\text {(syst)} \pm 6.0\,\text {(lumi)} \text { pb}.$ The result of an alternative analysis, which makes use of the observable $M_3$, is in good agreement with this value.

Furthermore, the analysis performed using data at $\sqrt{s}=7\,\text {TeV}$, yields a total cross section measurement of $161.7 \pm 6.0\,\text {(stat)} \pm 12.0\,\text {(syst)} \pm 3.6\,\text {(lumi)} \text { pb}$. The measured cross section ratio, where a number of experimental uncertainties cancel out, is $1.43 \pm 0.04\,\text {(stat)} \pm 0.07\,\text {(syst)} \pm 0.05\,\text {(lumi)}.$

All measurements are in agreement with the NNLO theoretical predictions.
